# Integrative Multivariate Genomics Identifies Shared Epithelial–Immune and Cytokine-Regulatory Mechanisms Across Major Chronic Lung Diseases

**DOI:** 10.3390/ijms27156946

**Published:** 2026-08-02

**Authors:** Chung-Chih Liao, Ke-Ru Liao, Jung-Miao Li

**Affiliations:** 1 School of Medicine, Chung Shan Medical University, Taichung 40201, Taiwan; cshy2232@csh.org.tw; 2 Department of Integrated Chinese and Western Medicine, Chung Shan Medical University Hospital, Taichung 40201, Taiwan; 3 Department of Neurology, Yuanlin Christian Hospital, Changhua 51052, Taiwan; u9401407@gmail.com; 4 School of Chinese Medicine, College of Chinese Medicine, China Medical University, Taichung 40402, Taiwan; 5 Department of Chinese Medicine, China Medical University Hospital, Taichung 40447, Taiwan

**Keywords:** chronic lung disease, genomic structural equation modeling, genome-wide association study, fine-mapping, transcriptomic prioritization, respiratory genetics, genetic pleiotropy

## Abstract

Chronic lung diseases, including asthma, chronic obstructive pulmonary disease, bronchiectasis, and idiopathic pulmonary fibrosis, are clinically distinct but share epithelial injury, host-defense, inflammatory, and remodeling processes. We integrated European-ancestry genome-wide association study (GWAS) summary statistics for these four diseases using genomic structural equation modeling to construct a multivariate chronic lung disease (mvCLD) factor. Variant-level association testing was combined with genomic control assessment, locus annotation, GWAS-by-subtraction, fine-mapping, transcriptomic prioritization, pathway enrichment, single-cell spatial mapping, and heritability partitioning. For discovery, across 6,255,777 autosomal variants, mvCLD identified 2067 genome-wide significant variants, 30 loci, and 53 lead variants. Five lead variants were genome-wide significant for mvCLD but not for any component disease and showed high-confidence fine-mapping support. For gene prioritization, transcriptomic and gene-level analyses prioritized 21 candidate genes, including *ORMDL3*, *GSDMB*, *IL18RAP*, *IL18R1*, *IL1R1*, *SMAD3*, and *CLEC16A*. In exploratory functional analyses, enrichment analyses converged on interleukin, cytokine-receptor, JAK-STAT, interleukin-4/interleukin-13, thymic stromal lymphopoietin, asthma, and lung fibrosis pathways. Exploratory single-cell spatial mapping, based on a mouse embryonic atlas, showed the strongest overall enrichment in the lung annotation, although cross-dataset cell-type and tissue analyses did not reach significance after false discovery rate correction; heritability partitioning implicated conserved and active regulatory elements. These findings support a shared epithelial–immune and cytokine-regulatory genetic architecture across major chronic lung diseases and nominate biologically coherent candidate genes and pathways for future functional and translational studies.

## 1. Introduction

Chronic lung diseases (CLDs) remain a major source of premature mortality, long-term disability, and health-care use worldwide [1]. Major CLD phenotypes include asthma, chronic obstructive pulmonary disease (COPD), bronchiectasis, and idiopathic pulmonary fibrosis (IPF), which together represent inflammatory airway disease, persistent airflow limitation, structural airway damage, and fibrotic interstitial lung disease, respectively. Asthma is characterized by variable airflow limitation and airway inflammation [2]; COPD is defined by persistent respiratory symptoms and incompletely reversible airflow limitation [3]; bronchiectasis reflects irreversible bronchial dilatation with impaired mucus clearance and recurrent infection [4]; and IPF represents a progressive fibrotic interstitial lung disease with poor prognosis [5]. These conditions are usually studied and treated as distinct disorders, yet in practice they often coexist, overlap in symptoms, and share partially convergent biological processes.

The boundaries between CLD phenotypes are therefore biologically informative but not absolute. Across chronic respiratory diseases, recurrent pathogenic axes include airway epithelial injury, epithelial barrier dysfunction, impaired host-defense responses, chronic immune activation, mucus dysregulation, and tissue remodeling [6]. In asthma, epithelial barrier disruption, impaired innate defense, persistent type 2 inflammation, and airway remodeling contribute to variable airflow limitation and disease heterogeneity [7]. In COPD, susceptibility and progression reflect lifelong interactions among genetic predisposition, environmental exposures, impaired lung growth, chronic airway inflammation, emphysema, and small-airway disease [8]. In bronchiectasis, impaired mucociliary clearance, persistent infection, dysregulated inflammation, and progressive structural airway damage form self-perpetuating disease cycles [9]. In IPF, repeated alveolar epithelial injury and aberrant repair responses promote fibroblast activation, extracellular matrix deposition, and progressive architectural distortion [10]. These shared pathogenic themes suggest that a subset of genetic risk may operate across conventional disease labels rather than within a single diagnosis alone.

Large-scale genome-wide association studies (GWASs) have substantially advanced the genetic understanding of CLD, although the depth of evidence differs across phenotypes [11]. In asthma, multi-ancestry GWASs have identified numerous susceptibility loci, including signals enriched near immune-cell regulatory elements, and have demonstrated that larger and more ancestrally diverse biobank datasets improve locus discovery and polygenic prediction [12,13]. For COPD and lung function, large-scale GWASs have expanded the catalog of association signals and highlighted genetic heterogeneity across ancestry groups, smoking-related strata, lung-function indices, cell types, and clinical definitions [14,15]. By contrast, bronchiectasis-specific genomic evidence remains comparatively limited; recent genomic and pathophysiological reviews emphasize host defense, epithelial biology, infection susceptibility, mucociliary dysfunction, and inflammatory regulation as key axes for bronchiectasis endotyping [9]. In IPF, GWASs have identified multiple susceptibility loci and reinforced the relevance of host defense, epithelial repair, telomere biology, and fibrotic remodeling pathways [16]. Importantly, CLD phenotypes are not genomically isolated: COPD susceptibility loci partly overlap with loci for lung function and pulmonary fibrosis, whereas asthma–COPD overlap studies indicate that shared and disease-specific genetic components can coexist within obstructive airway disease [17,18]. These findings support the need to move beyond single-disease GWASs and systematically evaluate whether a shared genetic architecture underlies clinically distinct CLD phenotypes.

Despite these advances, most genetic studies of CLD have remained anchored to single-disease endpoints, which has enabled disease-specific locus discovery but may not fully capture genetic effects distributed across related phenotypes [9,11,12,13,14,15,16,17,18]. Such effects may be attenuated by phenotypic heterogeneity, diagnostic misclassification, or differences in clinical ascertainment. This limitation is particularly relevant to CLD, in which diagnostic labels can be influenced by age, smoking exposure, infection history, lung-function thresholds, imaging availability, and health-care coding practices [9,10,13,15,18]. Moreover, analytical strategies that condition one respiratory phenotype on another may remove biologically meaningful shared liability, rather than simply isolating disease-specific genetic effects. A framework that models shared genetic covariance directly is therefore needed to complement conventional single-trait GWAS analyses.

Genomic structural equation modeling (Genomic SEM) provides such a framework by estimating the joint genetic architecture of correlated traits from summary-level GWAS data and defining latent genetic factors that capture shared liability across phenotypes [19]. In the present study, we used Genomic SEM to construct a multivariate chronic lung disease (mvCLD) factor from GWAS summary statistics for asthma, COPD, bronchiectasis, and IPF. We then organized the downstream analyses into two complementary evidence layers: first, variant-level discovery to identify mvCLD-specific lead variants that were not genome-wide significant in the component disease GWASs; and second, gene-level and functional genomic prioritization to determine whether the broader mvCLD association landscape converged on coherent respiratory biological contexts. This design was intended to clarify both the discovery value of multivariate modeling and the biological architecture shared across clinically distinct CLD phenotypes.

## 2. Results

### 2.1. Study Design and Analytical Workflow

**Figure 1:**
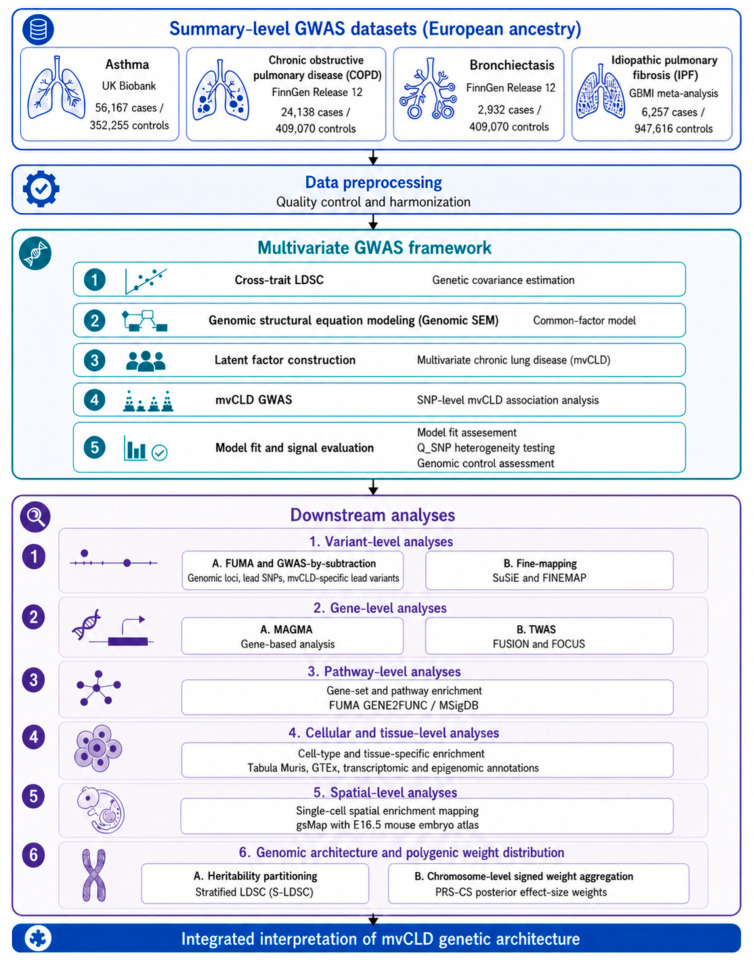
Study design and analytical workflow. Summary-level genome-wide association study (GWAS) datasets for asthma, chronic obstructive pulmonary disease, bronchiectasis, and idiopathic pulmonary fibrosis were harmonized and analyzed using Genomic SEM to construct mvCLD. Downstream analyses included variant-level, gene-level, pathway-level, cellular and tissue-level, spatial-level, and genomic architecture analyses. The vertical arrows denote a sequential backbone in which each step uses the output of the preceding step (univariate GWAS to quality control and harmonization to cross-trait LDSC to Genomic SEM to the mvCLD latent factor to the mvCLD GWAS to model evaluation). The mvCLD GWAS summary statistics are the shared input to the downstream analyses, which are largely parallel branches rather than a strict 1-to-6 sequence; within these branches, FUMA lead SNPs feed fine-mapping and the GWAS-by-subtraction comparison, the MAGMA gene-based results feed the pathway and cell-type/tissue enrichment, and the TWAS results feed FOCUS, whereas the gsMap spatial mapping, stratified-LDSC heritability partitioning, and PRS-CS analysis each use the mvCLD GWAS directly and independently. Abbreviations: COPD, chronic obstructive pulmonary disease; FUMA, Functional Mapping and Annotation of Genome-Wide Association Studies; GBMI, Global Biobank Meta-analysis Initiative; Genomic SEM, genomic structural equation modeling; GWAS, genome-wide association study; IPF, idiopathic pulmonary fibrosis; mvCLD, multivariate chronic lung disease; PRS-CS, polygenic risk score-continuous shrinkage; SNP, single-nucleotide polymorphism.

The overall study design and analytical workflow are summarized in Figure 1. Summary-level GWAS statistics for asthma, COPD, bronchiectasis, and IPF (Table 1) were harmonized and integrated using Genomic SEM to construct the multivariate chronic lung disease (mvCLD) latent factor, which was then carried forward through variant-level, gene-level, pathway-level, cellular and tissue-level, spatial-level, and genomic-architecture analyses. To make the logical sequence and data flow explicit, the workflow followed a sequential backbone in which each step used the output of the previous step: the four univariate GWAS were quality-controlled and harmonized; cross-trait LDSC produced the genetic covariance matrix; Genomic SEM fitted the common-factor model to define the mvCLD latent factor; and SNP-level association testing generated the mvCLD GWAS summary statistics, which were then evaluated for model fit, SNP-level heterogeneity (Q_SNP), and genomic control. The mvCLD GWAS summary statistics then served as the shared input for the downstream analyses, which were largely parallel branches rather than a single linear sequence. Within these branches, some analyses used the output of an earlier analysis as input, whereas others were independent: the FUMA-defined loci and lead SNPs provided the regions for fine-mapping (SuSiE and FINEMAP) and the variants for the GWAS-by-subtraction comparison; the MAGMA gene-based results provided the gene input for both the GENE2FUNC pathway enrichment and the cell-type and tissue-specific enrichment; and the TWAS (FUSION) results defined the regions subsequently fine-mapped with FOCUS, after which MAGMA, TWAS, and FOCUS were integrated to prioritize candidate genes. By contrast, the single-cell spatial mapping (gsMap), the stratified-LDSC heritability partitioning, and the PRS-CS polygenic-weight analysis each took the mvCLD GWAS summary statistics directly and were independent of the gene-level and variant-level branches. These input–output relationships correspond to the top-to-bottom structure shown in Figure 1.

### 2.2. Construction of the mvCLD Shared Genetic Factor

**Figure 2:**
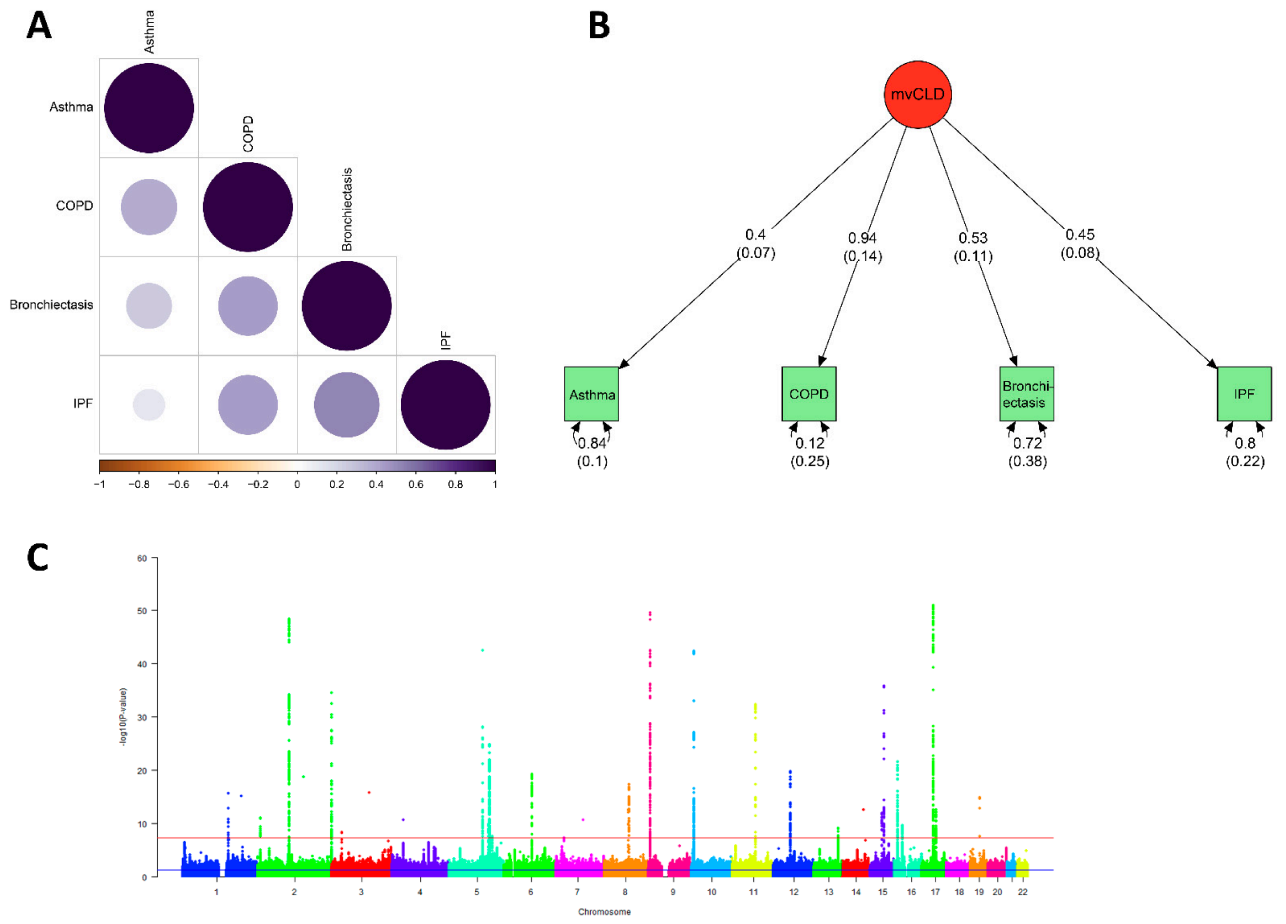
Genomic SEM analysis and SNP-level association results for mvCLD. (**A**) Genetic correlation matrix among asthma, chronic obstructive pulmonary disease, bronchiectasis, and idiopathic pulmonary fibrosis estimated using linkage disequilibrium score regression. (**B**) Single-factor Genomic SEM model showing standardized factor loadings for the four chronic lung disease traits; standard errors are shown in parentheses. (**C**) Manhattan plot showing genome-wide SNP associations with mvCLD. The x-axis represents chromosomal position, and the y-axis represents −log_10_(*p*) of SNP-level association *p* values. The horizontal red dashed line denotes the conventional genome-wide significance threshold, *p* = 5 × 10^−8^. Abbreviations: COPD, chronic obstructive pulmonary disease; Genomic SEM, genomic structural equation modeling; IPF, idiopathic pulmonary fibrosis; mvCLD, multivariate chronic lung disease; SNP, single-nucleotide polymorphism.

LDSC analysis showed that the four univariate input GWAS datasets, including asthma, COPD, bronchiectasis, and IPF, had estimable SNP heritability (h^2^), with estimates of 0.047, 0.038, 0.003, and 0.120, respectively (Supplementary Table S1). Overall positive genetic correlations were observed among the four CLD phenotypes. For asthma, the pairwise genetic correlations with COPD, bronchiectasis, and IPF were 0.387, 0.257, and 0.123, respectively (Supplementary Table S2; Figure 2A).

Based on these LDSC estimates, we fitted a single common-factor model to evaluate whether the four input GWAS datasets could be represented by a shared genetic factor. Genomic SEM showed that the common-factor model had acceptable fit (CFI = 0.985; SRMR = 0.096; Supplementary Table S3; Figure 2B). In the standardized estimates, the factor loadings of the latent factor F1 on asthma, COPD, bronchiectasis, and IPF were 0.403, 0.940, 0.527, and 0.452, respectively (Supplementary Table S4). All six pairwise genetic correlations among the four phenotypes were positive (range 0.123 to 0.527; Supplementary Table S2), and COPD showed the highest average pairwise genetic correlation with the other three phenotypes, supporting a single shared liability dimension rather than two or more independent trait clusters. All four standardized loadings were positive and highly significant (asthma *p* = 2.25 × 10^−9^; COPD *p* = 4.61 × 10^−12^; bronchiectasis *p* = 1.12 × 10^−6^; IPF *p* = 4.30 × 10^−8^; Supplementary Table S4), and the single-factor model was not rejected by the model chi-square test (χ^2^ = 5.26, df = 2, *p* = 0.072; Supplementary Table S3). Although COPD showed the highest loading, indicating that it aligned most closely with the shared factor and reflecting its central position in the genetic-correlation network, the model retained substantial disease-specific residual variance for asthma, bronchiectasis, and IPF (standardized residual variance 0.84, 0.72, and 0.80, respectively; Supplementary Table S4), indicating that these phenotypes were not collapsed into a single COPD-defined axis. These results indicate that the four CLD phenotypes can be represented by a shared genetic construct, which we defined as mvCLD.

### 2.3. SNP-Level mvCLD Association Analysis

After confirming the single common-factor model, we extended the SEM framework to the SNP level to construct multivariate GWAS results for mvCLD. This analysis evaluated associations between 6,255,777 SNPs and the mvCLD latent factor (Figure 2C).

At the genome-wide significance threshold (*p* < 5 × 10^−8^), 2067 SNPs were significantly associated with mvCLD (Supplementary Table S5). At more stringent thresholds, 1409 SNPs reached *p* < 5 × 10^−12^, of which 916 further reached *p* < 5 × 10^−16^ (Supplementary Table S6). The strongest association signal was rs2305479 (β = 0.054, standard error (SE) = 0.004, *p* = 1.17 × 10^−51^), located at 17q12. Examination of the Genomic SEM Q_SNP statistic indicated residual heterogeneity for a subset of mvCLD-associated signals across the four component CLD phenotypes, suggesting that the mvCLD association results included both broadly shared effects and signals with partially disease-skewed contributions. Therefore, Q_SNP was interpreted as an auxiliary diagnostic metric rather than as an exclusion criterion for mvCLD-associated variants. These SNP-level mvCLD results served as the primary input for subsequent genomic locus annotation, fine-mapping, and functional analyses.

### 2.4. Genomic Control Evaluation of mvCLD GWAS Based on LDSC

To evaluate potential systematic statistical inflation in the mvCLD GWAS results, we performed LDSC-based genomic control analysis. The original mvCLD GWAS summary statistics included 6,255,777 SNPs. After matching to the HapMap3 SNP reference list and allele harmonization, 947,256 SNPs were retained for LDSC analysis. After merging with the European LD score reference data, 944,907 SNPs were included in the two-step LDSC estimation.

The mvCLD GWAS had a mean χ^2^ statistic of 1.211, λGC of 1.287, and maximum χ^2^ statistic of 228.657. The observed-scale SNP heritability was 0.021 (SE = 0.001), and the LDSC intercept was 0.895 (SE = 0.007). These genomic control metrics did not indicate substantial genome-wide statistical inflation attributable to systematic bias.

### 2.5. mvCLD Locus Discovery and mvCLD-Specific Lead Variants

**Figure 3:**
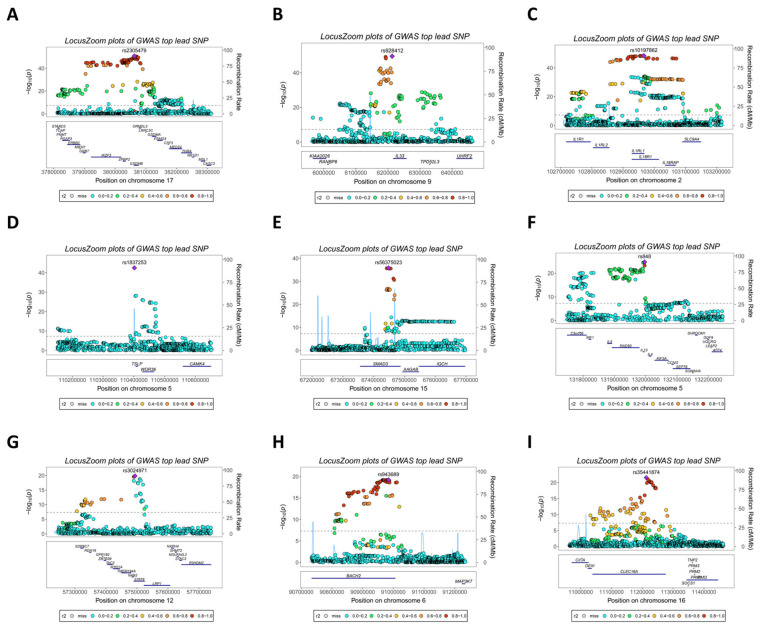
Regional association plots for nine representative mvCLD-associated loci. LocusZoom plots illustrating regional association signals for nine representative loci prioritized from the mvCLD GWAS using FUMA. Panels (**A**–**I**) show locus-specific association patterns. The x-axis indicates genomic position, and the y-axis represents −log_10_(*p*) of SNP association *p* values. The purple diamond indicates the lead SNP in each locus. Other variants are color-coded according to linkage disequilibrium with the lead SNP, expressed as *r*^2^, as shown in the legend. Gene annotation tracks are displayed below each plot. The horizontal dashed line denotes the genome-wide significance threshold, *p* = 5 × 10^−8^. These loci were selected to represent prominent regional association patterns across the mvCLD GWAS results. Abbreviations: FUMA, Functional Mapping and Annotation of Genome-Wide Association Studies; GWAS, genome-wide association study; LD, linkage disequilibrium; mvCLD, multivariate chronic lung disease; SNP, single-nucleotide polymorphism.

To characterize genome-wide significant signals identified in the mvCLD GWAS, we used FUMA for genomic locus definition, lead SNP annotation, and gene-based association analysis. According to FUMA-defined criteria, 30 mvCLD-associated genomic loci were identified, including 177 independent significant SNPs and 53 lead SNPs (Supplementary Table S7; Figure 3). To allow direct inspection of how each lead variant behaves in the individual component diseases, the disease-specific effect estimates, standard errors, and *p* values for all 53 mvCLD lead SNPs in each of the four component GWASs (asthma, COPD, bronchiectasis, and IPF) are provided in Supplementary Table S10, not only for the five mvCLD-specific variants highlighted in Table 2. MAGMA gene-based analysis further identified 136 genes significantly associated with mvCLD (false discovery rate [FDR] < 0.05; Supplementary Table S8), including *IL1RL1*, *IL18RAP*, *TSLP*, *IL33*, *SMAD3*, *STAT6*, and *GSDMB*.

Functional annotation of the 53 lead SNPs showed that these variants were mainly located in noncoding regions, particularly intergenic and intronic regions (Supplementary Table S9). The most significant lead SNP was rs2305479, located at 17q12 near *GSDMB* (β = 0.054, SE = 0.004, *p* = 1.17 × 10^−51^).

Using the GWAS-by-subtraction strategy, we identified five mvCLD-specific lead SNPs after comparing mvCLD lead SNPs with the four original univariate GWAS datasets: rs72736978, rs71208329, rs11922517, rs4562243, and rs118084239 (Table 2; Supplementary Table S10). These variants reached genome-wide significance in the mvCLD GWAS but not in any univariate GWAS for asthma, COPD, bronchiectasis, or IPF, indicating that they were detected through the multivariate framework. The remaining 48 lead SNPs overlapped with at least one original univariate GWAS signal. For each of these five variants, the disease-specific effect estimates, standard errors, *p* values, effect-allele frequencies, and effect directions in all four component GWASs are reported in Supplementary Table S10 and are displayed as forest plots in Supplementary Figure S1. Consistent with their operational definition, none of the five variants reached genome-wide significance in any single component GWAS, and in the datasets in which they were adequately estimated (asthma and IPF) their effects were close to the null. All five variants passed the minor-allele-frequency quality-control threshold of 0.01 that was applied to the harmonized analysis allele frequency (analysis minor allele frequency 0.013 to 0.439; Supplementary Table S10). Four of the five were low-frequency overall (analysis minor allele frequency approximately 0.013 to 0.043), whereas rs4562243 was common overall (analysis minor allele frequency approximately 0.439). In the FinnGen COPD and bronchiectasis summary statistics, however, the effect-allele frequency of all five variants was very low (<0.001; Supplementary Table S10), well below the 0.01 threshold used for the multivariate analysis; consequently, these variants are uninformative in the FinnGen-based component traits, and their COPD- and bronchiectasis-specific values in Supplementary Table S10, which represent look-ups against the original univariate summary statistics for the GWAS-by-subtraction comparison, are correspondingly imprecise. The term “mvCLD-specific” is therefore used in a strictly operational sense, denoting sub-threshold significance in each univariate GWAS within the present framework; it does not, in the absence of external comparison and functional follow-up, imply biological specificity, novelty relative to other studies, or causal relevance, and these variants require replication in independent datasets before any such interpretation.

### 2.6. Fine-Mapping

To identify candidate variants with high posterior support within mvCLD-associated loci, we integrated SuSiE and FINEMAP fine-mapping results. Using SuSiE posterior probability ≥ 0.950 and FINEMAP posterior probability ≥ 0.950 as the high-confidence threshold, 18 distinct SNPs met this criterion, corresponding to 27 candidate variant-gene annotation entries (Supplementary Table S11). Among these, 12 distinct SNPs were further classified as consensus SNPs, reflecting concordant credible-set support across SuSiE and FINEMAP.

Among the high-confidence candidate variants, eight distinct SNPs were also mvCLD GWAS lead SNPs: rs1837253, rs34290285, rs71208329, rs11922517, rs12123821, rs72736978, rs118084239, and rs4562243. The five mvCLD-specific lead SNPs described above (rs72736978, rs71208329, rs11922517, rs4562243, and rs118084239) were all included in this high-confidence set. For every high-confidence variant, the corresponding SuSiE 95% credible set comprised a single variant, with essentially all of the regional posterior inclusion probability concentrated on that variant. The posterior probabilities approaching 1.000 therefore reflect these very small, single-variant credible sets rather than implausible certainty across a broad linkage disequilibrium (LD) block. Because fine-mapping posterior probabilities are conditional on the LD reference panel and on the assumed number of causal variants, they were interpreted as strong statistical prioritization rather than definitive causal assignment, and the concordance between SuSiE and FINEMAP, which use different priors, reduces but does not fully exclude the possibility of LD-driven artifacts.

**Figure 4:**
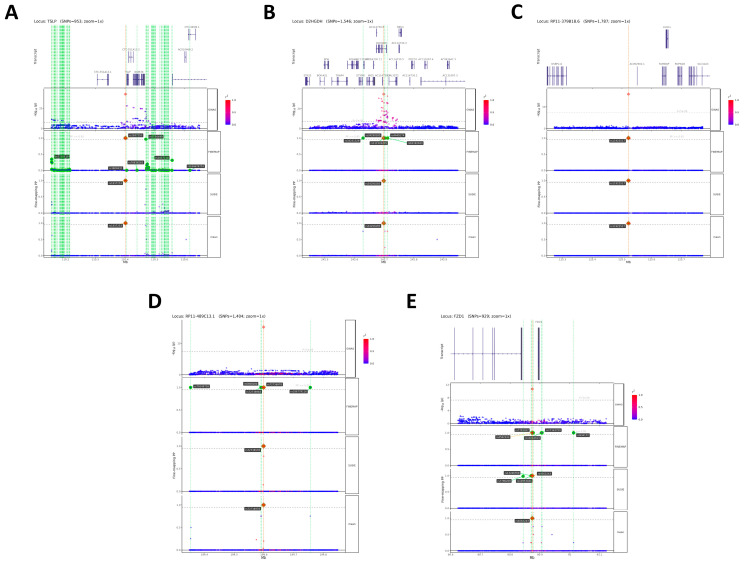
Fine-mapping results for representative high-confidence mvCLD loci. Panels (**A**–**E**) display locus-specific fine-mapping views for the *TSLP*/*WDR36*, *D2HGDH*, *RP11-379B18.6*, *RP11-489C13.1*, and *FZD1* loci, respectively. For the *TSLP*/*WDR36* region, the *TSLP*-centered plot is shown because rs1837253 represents the shared fine-mapping signal across this locus. These loci were selected because their corresponding variants were simultaneously identified as mvCLD lead SNPs and consensus SNPs and showed high posterior support in both SuSiE and FINEMAP analyses. For each locus, the tracks display, from top to bottom: gene models; GWAS association signals, with the y-axis representing −log_10_(*p*); posterior inclusion probability derived from FINEMAP; posterior inclusion probability derived from SuSiE; and mean posterior inclusion probability across both fine-mapping methods. The x-axis represents chromosomal position in megabases. Lead SNPs are highlighted by vertical dashed lines, and variants are color-coded according to linkage disequilibrium with the top signal, expressed as *r*^2^. Abbreviations: FINEMAP, fine-mapping software package; GWAS, genome-wide association study; mvCLD, multivariate chronic lung disease; SNP, single-nucleotide polymorphism; SuSiE, Sum of Single Effects.

Focusing further on variants meeting both lead SNP and consensus SNP criteria, five distinct SNPs showed the most consistent cross-method support: rs1837253, rs34290285, rs11922517, rs72736978, and rs4562243. These variants mapped to the *TSLP*/*WDR36*, *D2HGDH*, *RP11-379B18.6*, *RP11-489C13.1*, and *FZD1* regions, respectively. Representative regional fine-mapping results are shown in Figure 4.

### 2.7. Transcriptomic Gene Prioritization of the Broader mvCLD Architecture

**Figure 5:**
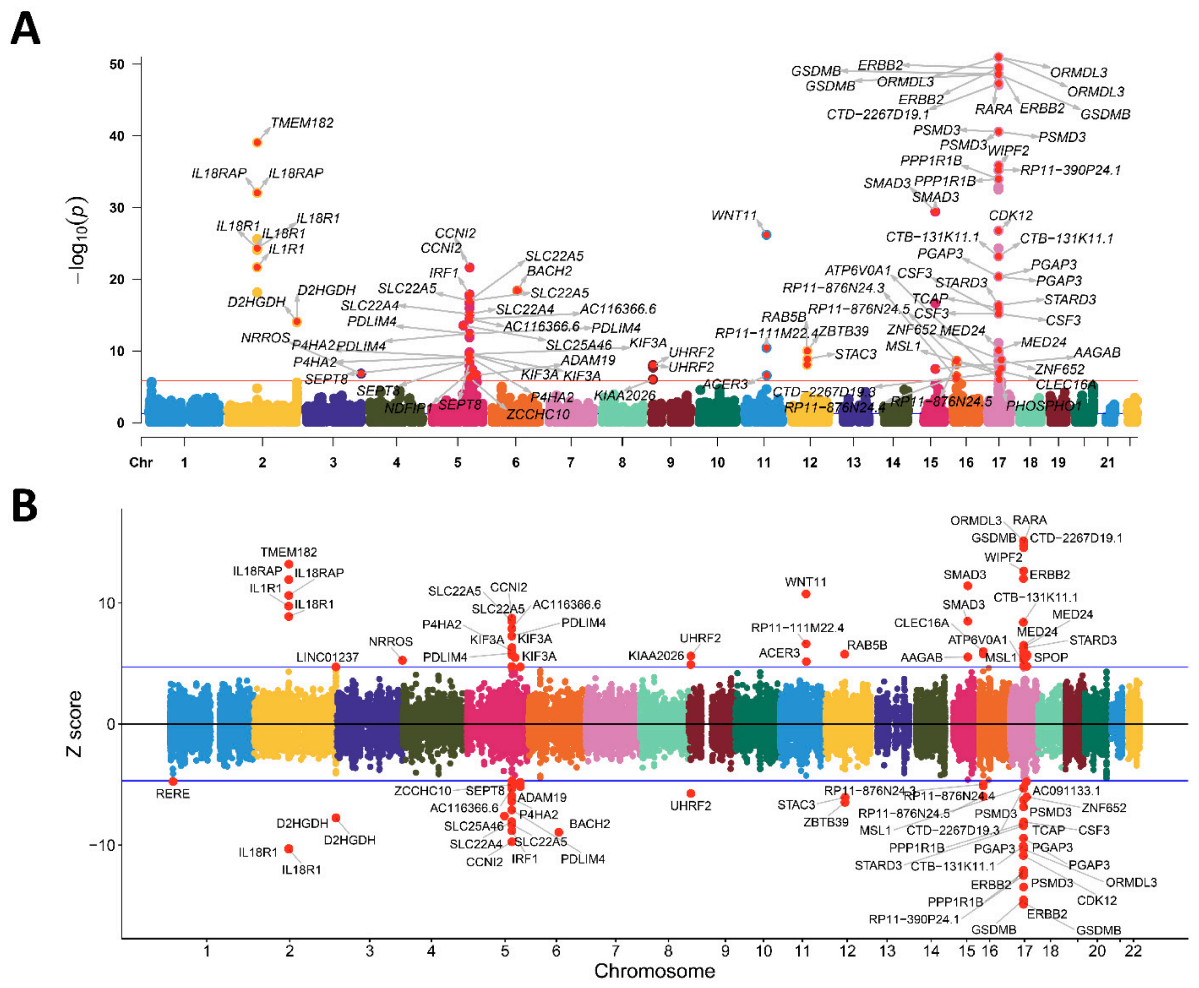
Transcriptome-wide association study results for mvCLD. (**A**) Manhattan plot showing gene-level transcriptome-wide association study associations across chromosomes using FUSION. The x-axis represents chromosomal position, and the y-axis represents −log_10_(*p*) of transcriptome-wide association study *p* values. The horizontal red line denotes the Bonferroni-corrected transcriptome-wide significance threshold, *p* = 1.319 × 10^−6^, whereas the horizontal blue line indicates the nominal significance threshold, *p* = 0.05. Representative genes with prominent transcriptome-wide association signals are labeled. (**B**) Manhattan plot showing transcriptome-wide association study *Z*-scores across chromosomes. The y-axis represents standardized *Z*-scores, where positive and negative values indicate the direction of association between genetically predicted gene expression and mvCLD. The horizontal blue lines denote the prespecified visualization threshold, |*Z*| = 4.697. Abbreviations: FUSION, Functional Summary-based Imputation; mvCLD, multivariate chronic lung disease.

We performed TWAS using FUSION to identify gene-level transcriptomic associations related to the broader mvCLD genetic architecture. After Bonferroni correction (*p* < 1.319 × 10^−6^), 92 transcriptome-wide significant gene-expression weight associations were identified, corresponding to 55 distinct genes (Supplementary Table S12; Figure 5). The strongest TWAS signals were concentrated in major mvCLD GWAS regions, particularly 17q12-q21 and 2q12. The 17q12-q21 region included *ORMDL3*, *RARA*, *ERBB2*, *GSDMB*, and *PSMD3*, whereas the 2q12 region included *IL18RAP*, *IL18R1*, and *IL1R1*. The strongest TWAS signals were observed for *ORMDL3* (*p* = 1.08 × 10^−51^), *RARA* (*p* = 2.78 × 10^−50^), *ERBB2* (*p* = 3.65 × 10^−50^), and *GSDMB* (*p* = 2.67 × 10^−49^). These transcriptomic findings prioritized genes within the overall mvCLD association landscape and should not be interpreted as direct one-to-one target assignment for the five mvCLD-specific lead SNPs.

We then used FOCUS to perform gene-level fine-mapping of TWAS-significant regions. After excluding NULL model entries from the FOCUS output, 67 candidate gene results had PIP > 0.500, corresponding to 50 distinct genes; among these, 40 results met the high-confidence threshold of PIP > 0.950, corresponding to 34 distinct genes (Supplementary Table S13).

After integrating TWAS significance with FOCUS posterior evidence, 26 high-confidence gene-expression weight associations were identified, corresponding to 21 distinct candidate genes (Table 3; Supplementary Table S14). These genes included *ORMDL3*, *GSDMB*, *TMEM182*, *IL18RAP*, *SMAD3*, *WNT11*, *IL18R1*, *IL1R1*, *CCNI2*, *BACH2*, *IRF1*, *SLC22A5*, *D2HGDH*, *SLC25A46*, *CLEC16A*, *RAB5B*, *UHRF2*, *NDFIP1*, *ZNF652*, *STAC3*, and *KIAA2026*. Table 3 summarizes these genes by regional GWAS signal, FOCUS-supported cross-tissue sCCA feature, TWAS direction, FOCUS posterior support, and MAGMA gene-based support.

### 2.8. Pathway Enrichment Analysis

MAGMA gene-based analysis identified 65 genes significantly associated with mvCLD using a Bonferroni-corrected *p* value < 0.05 (Supplementary Table S15). These genes included *IL1R1*, *IL1RL1*, *IL18R1*, *IL18RAP*, *TSLP*, *WDR36*, *IL5*, *IL13*, *IL4*, *STAT6*, *SMAD3*, *IL4R*, *GSDMB*, *ORMDL3*, and *CLEC16A* (Supplementary Table S15). Several TWAS/FOCUS-prioritized genes also reached Bonferroni-corrected significance in MAGMA gene-based analysis, including ORMDL3, GSDMB, IL18RAP, IL18R1, IL1R1, SMAD3, CCNI2, BACH2, IRF1, SLC22A5, D2HGDH, CLEC16A, RAB5B, NDFIP1, and ZNF652 (Table 3; Supplementary Table S15).

Using these MAGMA-significant genes for FUMA GENE2FUNC enrichment analysis, 545 gene sets reached FDR significance (FDR-corrected *p* value < 0.05; Supplementary Table S16). In the canonical pathway analysis, significantly enriched terms included Reactome signaling by interleukins (FDR-corrected *p* = 3.759 × 10^−13^), Reactome cytokine signaling in immune system (FDR-corrected *p* = 4.090 × 10^−11^), Reactome interleukin-1 family signaling (FDR-corrected *p* = 3.686 × 10^−8^), Kyoto Encyclopedia of Genes and Genomes (KEGG) JAK-STAT signaling pathway (FDR-corrected *p* = 3.836 × 10^−8^), KEGG cytokine–cytokine receptor interaction (FDR-corrected *p* = 1.824 × 10^−7^), and Reactome interleukin-18 signaling (FDR-corrected *p* = 1.454 × 10^−6^).

Additional significant enrichment terms included Reactome interleukin-4 and interleukin-13 signaling (FDR-corrected *p* = 0.002), WikiPathways lung fibrosis (FDR-corrected *p* < 0.001), WikiPathways thymic stromal lymphopoietin signaling pathway (FDR-corrected *p* = 0.025), KEGG asthma (FDR-corrected *p* = 0.005), and KEGG Fc epsilon RI signaling pathway (FDR-corrected *p* = 0.007). GO enrichment terms included regulation of immunoglobulin production, leukocyte differentiation, cell activation, positive regulation of cytokine production, and regulation of immune system process. GWAS Catalog trait enrichment included asthma, asthma or allergic disease pleiotropy, childhood-onset asthma, adult-onset asthma, allergic rhinitis, eosinophil counts, and moderate/severe asthma. These enrichment results are partly influenced by well-characterized, highly pleiotropic and asthma-associated loci, including 17q12-q21, the 2q12 interleukin-1 receptor cluster, the thymic stromal lymphopoietin (5q22) region, and the 5q31 interleukin-4/interleukin-13 cluster. To assess the extent of this influence, we examined the genomic distribution of the 136 MAGMA-significant genes that served as the input to the gene-set enrichment analysis. Of these 136 genes, 42 (31%) were located within the four regions noted above (17q12-q21, *n* = 19; 2q12, n = 6; 5q22/thymic stromal lymphopoietin, n = 3; 5q31, n = 14), whereas the remaining 94 genes (69%) were distributed across 17 different chromosomes. The genes outside these four regions included multiple canonical immune and cytokine-signaling genes, such as STAT6, SMAD3, BACH2, CLEC16A, RORA, and RUNX3, that contribute to the enriched interleukin, cytokine-receptor, and JAK-STAT terms independently of the four named loci. This distribution indicates that the enrichment signal is supported by genes spread broadly across the genome rather than being attributable to a small number of pleiotropic regions, although we did not perform a formal re-estimation of enrichment after excluding these loci, and the enrichment terms should therefore be interpreted as influenced by, but not solely driven by, these well-known asthma-associated regions.

### 2.9. Cross-Dataset Cell-Type and Tissue-Specific Enrichment Analysis

To evaluate whether mvCLD gene-level signals showed cell-type, tissue, or epigenomic annotation specificity, we integrated Tabula Muris single-cell data, GTEx tissue expression data, multitissue transcriptomic annotations, and multitissue epigenomic annotations for enrichment analysis. No annotation reached FDR-corrected significance in any of the four datasets (FDR-corrected *p* value < 0.05; Supplementary Tables S17–S20). Therefore, the following results should be interpreted as nominal-level exploratory trends.

In the Tabula Muris single-cell analysis, the leading nominal enrichment signals included liver natural killer cell (*p* = 0.003, FDR = 0.278), limb muscle T cell (*p* = 0.007, FDR = 0.278), marrow immature T cell (*p* = 0.011, FDR = 0.278), skin epidermal cell (*p* = 0.012, FDR = 0.278), lung T cell (*p* = 0.016, FDR = 0.278), and trachea mesenchymal cell (*p* = 0.017, FDR = 0.278) (Supplementary Table S17).

In the human GTEx tissue-level analysis, no tissue reached FDR significance. Whole blood showed the leading nominal signal (*p* = 0.026, FDR = 0.666), whereas lung did not reach nominal significance (*p* = 0.080, FDR = 0.708) (Supplementary Table S18). In the human multitissue transcriptomic annotation analysis, nominal-level signals were dispersed and included non-respiratory annotations as well as T lymphocytes, regulatory T lymphocytes, CD4-positive T lymphocytes, lymphocytes, and whole blood; all annotations failed to pass FDR correction (Supplementary Table S19). In the human multitissue epigenomic annotation analysis, the leading nominal signals included primary T helper 17 cells PMA-I stimulated H3K4me1, primary T helper memory cells H3K4me1, primary T helper cells PMA-I stimulated H3K27ac, and primary T cells from peripheral blood H3K27ac, but none reached FDR significance (Supplementary Table S20).

Overall, the cross-dataset cell-type and tissue-specific enrichment analyses did not identify robust enrichment for any cell-type, tissue, or epigenomic annotation; nominal-level signals were treated as descriptive results.

### 2.10. Single-Cell Spatial Enrichment Signatures of mvCLD Genetic Signals

**Figure 6:**
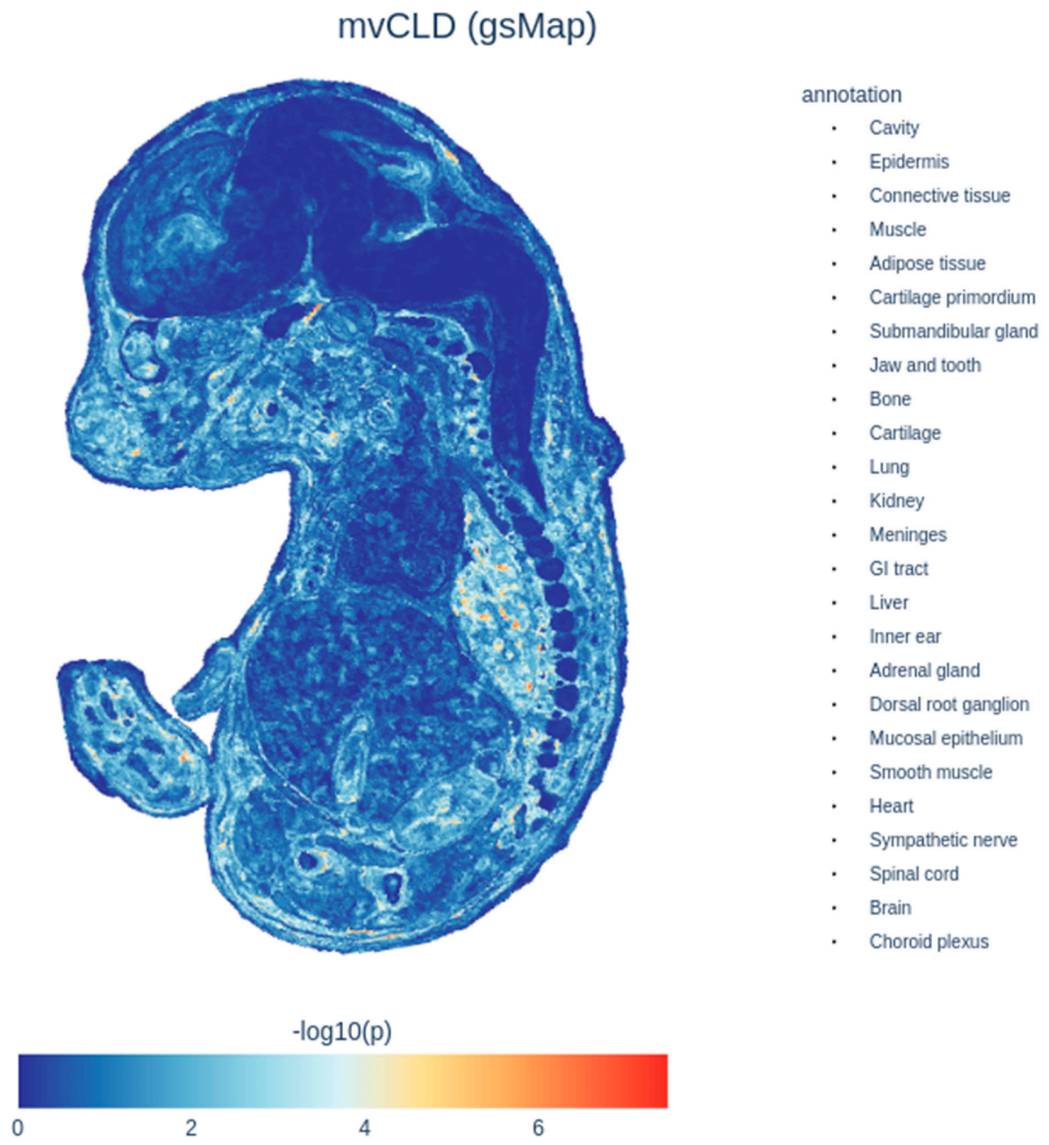
Single-cell spatial enrichment map of mvCLD genetic signals using gsMap. The spatial enrichment map shows the projection of mvCLD GWAS-derived polygenic signals onto a single-cell spatial transcriptomic atlas of a mouse embryo at embryonic day 16.5. The color gradient represents spatial enrichment significance expressed as −log_10_(*p*), with warmer colors indicating stronger local enrichment signals. Anatomical annotations of the tissue regions included in the embryonic day 16.5 atlas are listed on the right. The strongest overall spatial enrichment pattern was observed in the lung annotation, with additional local signals in selected epithelial, connective tissue, cartilage-related, and other tissue regions. This analysis is exploratory: it projects human mvCLD signals onto a mouse embryonic (E16.5) developmental atlas, which is biologically distant from adult chronic lung disease, and the cross-dataset cell-type and tissue analyses did not reach FDR significance; the result is therefore hypothesis-generating and does not establish adult human lung cell-type localization. Abbreviations: E16.5, embryonic day 16.5; gsMap, spatial genetic mapping framework; GWAS, genome-wide association study; mvCLD, multivariate chronic lung disease.

We used gsMap to project mvCLD GWAS polygenic signals onto the E16.5 mouse embryo single-cell spatial transcriptomic atlas. The spatial enrichment map showed that mvCLD signals were not uniformly distributed across the embryo but instead showed locally increased −log_10_(*p*) signals in selected anatomical annotations (Figure 6; Supplementary Table S21).

Among anatomical annotations, lung showed the most concentrated overall enrichment signal, with a median *p* value of 0.005 and a corresponding median −log_10_(*p*) of 2.331; the local peak *p* value reached 9.72 × 10^−8^, corresponding to a maximum −log_10_(*p*) of 7.012. In addition to lung, several epithelial, mesenchymal, and developmental structural annotations showed higher spatial enrichment signals, including cartilage primordium (median *p* = 0.013; maximum −log_10_(*p*) = 7.438), mucosal epithelium (median *p* = 0.017; maximum −log_10_(*p*) = 6.601), kidney (median *p* = 0.017; maximum −log_10_(*p*) = 5.560), adipose tissue (median *p* = 0.022; maximum −log_10_(*p*) = 6.468), cartilage (median *p* = 0.027; maximum −log_10_(*p*) = 6.773), and connective tissue (median *p* = 0.034; maximum −log_10_(*p*) = 7.480).

By comparison, the median *p* values for brain, dorsal root ganglion, and spinal cord were 0.748, 0.753, and 0.891, respectively; the median *p* values for heart and liver were 0.518 and 0.243. Overall, gsMap spatial enrichment analysis showed a nonuniform spatial distribution of mvCLD genetic signals in the E16.5 mouse embryo atlas, with the most evident overall enrichment in the Lung annotation. This spatial analysis was exploratory. Because it projected human mvCLD signals onto a mouse embryonic (E16.5) developmental atlas, which is biologically distant from adult chronic lung disease, and because the cross-dataset cell-type and tissue analyses (Section 2.9) did not reach FDR significance, these results should be regarded as a hypothesis-generating anatomical signal rather than as evidence of adult human lung cell-type or disease-stage localization, and would require validation in adult human lung single-cell or spatial datasets.

### 2.11. Heritability Partitioning Across Genomic Regions

To evaluate the distribution of mvCLD genetic signals across functional genomic regions, we performed S-LDSC heritability partitioning. Overall, multiple functional genomic annotations showed significant enrichment after FDR correction (FDR < 0.05; Supplementary Table S22).

Among all annotations, Conserved Lindblad-Toh covered only 2.6% of SNPs but explained 34.8% of mvCLD SNP heritability, corresponding to an enrichment of 13.553 (FDR = 2.385 × 10^−10^). Transcription start site and promoter-related regions were also significantly enriched, including TSS Hoffman (enrichment = 10.182; FDR = 2.827 × 10^−4^) and Promoter UCSC (enrichment = 4.937; FDR = 0.003). Protein-coding regions were also significantly enriched: Coding UCSC covered 1.4% of SNPs and explained 9.0% of SNP heritability, with an enrichment of 6.282 (FDR = 6.064 × 10^−4^).

Among noncoding regulatory annotations, Enhancer Andersson showed the highest enrichment (enrichment = 16.457; FDR = 0.025), while Enhancer Hoffman (enrichment = 6.347; FDR = 0.003) and its 500-bp extended region (enrichment = 4.540; FDR = 4.231 × 10^−5^) were also significant. Super-enhancer Hnisz and its extended region were also significant, with enrichments of 2.388 (FDR = 2.516 × 10^−11^) and 2.275 (FDR = 1.062 × 10^−11^), respectively. Open chromatin or transcription factor binding-related annotations, including DGF ENCODE, DHS Trynka, DHS peaks Trynka, and TFBS ENCODE, also reached FDR significance.

Active chromatin mark analysis showed FDR-significant enrichment for H3K27ac Hnisz, H3K27ac PGC2, H3K4me1 Trynka, H3K4me3 Trynka, and H3K9ac Trynka. Representative signals included H3K4me3 Trynka (enrichment = 4.014; FDR = 1.312 × 10^−6^), H3K9ac Trynka (enrichment = 3.479; FDR = 1.311 × 10^−4^), and H3K27ac PGC2 (enrichment = 2.843; FDR = 6.514 × 10^−8^). By contrast, Repressed Hoffman and its 500-bp extended region had enrichments of 0.523 (FDR = 0.038) and 0.566 (FDR = 5.255 × 10^−11^), respectively, indicating a lower heritability proportion than the corresponding SNP proportion.

### 2.12. Chromosome-Level Distribution of PRS-CS Posterior Effect-Size Weights

To describe the chromosome-level distribution of mvCLD polygenic effect-size weights, we summed PRS-CS posterior SNP effect sizes by chromosome. In total, 897,970 SNPs across 22 autosomes were included in the chromosome-level aggregation (Supplementary Table S23). The number of SNPs per chromosome ranged from 12,694 to 75,750, with chr2, chr1, and chr3 containing 75,750, 75,709, and 63,012 SNPs, respectively.

For the signed posterior effect-size weight sum, the largest positive aggregate was observed for chromosome 9 (weight sum = 0.097), followed by chromosome 7 (0.047), chromosome 15 (0.046), chromosome 22 (0.018), and chromosome 19 (0.004). The largest negative aggregate was observed for chromosome 18 (−0.135), followed by chromosome 2 (−0.126), chromosome 1 (−0.123), chromosome 20 (−0.119), chromosome 11 (−0.105), chromosome 4 (−0.100), and chromosome 3 (−0.091) (Supplementary Table S23). These chromosome-level signed sums are presented only as a descriptive summary of the genome-wide distribution of polygenic weights. Because the signed sum of posterior effect sizes depends on the arbitrary effect-allele coding, on local LD structure and variant density, and on cancellation between variants with opposing effect directions, its magnitude and sign do not carry a direct biological interpretation and should not be read as a measure of the disease relevance of individual chromosomes.

## 3. Discussion

This study identified a quantifiable shared genetic component across asthma, COPD, bronchiectasis, and IPF and used this component to define mvCLD. The central contribution of this work is not simply the enumeration of additional GWAS signals, but the construction of a multivariate respiratory genetic phenotype that captures a layer of inherited liability shared across obstructive, inflammatory, structural airway, and fibrotic lung disorders. The results support a two-level interpretation: mvCLD improves variant discovery by identifying lead variants not significant in individual disease GWASs, and the broader mvCLD association architecture converges on epithelial–immune and cytokine-regulatory biology. This structure provides a clearer framework for interpreting the subsequent analyses and avoids collapsing clinically distinct CLDs into a single disease entity.

A central question for any multivariate genetic phenotype is whether the latent factor reflects a genuinely shared liability or is instead dominated by a single component trait. Several lines of evidence argue against the interpretation that mvCLD is merely a proxy for COPD or for smoking- and lung-function-related liability. First, the four phenotypes were defined by clinical disease diagnoses rather than by smoking behavior or quantitative lung-function measures, and all six pairwise genetic correlations among them were positive, with all four standardized factor loadings positive and statistically significant; each phenotype therefore contributed to the shared factor rather than the factor being defined by COPD alone. The comparatively high COPD loading is consistent with COPD occupying the most central position in the genetic-correlation network, yet the model retained substantial disease-specific residual variance for asthma, bronchiectasis, and IPF, indicating that these phenotypes were not absorbed into a COPD-defined axis. Second, the biological content of the shared signal is not that expected of a smoking-, emphysema-, or lung-function-dominated liability. If mvCLD were driven primarily by such liability, its strongest signals would be expected to concentrate at canonical smoking-dependence and lung-function loci; instead, the leading mvCLD signals and the strongest enrichment results were dominated by epithelial-alarmin and type 2 inflammatory biology, including the 17q12-q21 (ORMDL3/GSDMB) locus, the 2q12 IL1RL1/IL18R1/IL18RAP region, and TSLP, IL33, and IL4/IL13/STAT6 signaling, with GWAS Catalog enrichment for asthma, allergic disease, and eosinophil-related traits. This profile is characteristic of a cross-cutting epithelial–immune axis rather than a COPD- or smoking-specific construct. Third, the SNP-level heterogeneity statistic (Q_SNP), applied as a variant-level sensitivity diagnostic of the common-factor assumption, flagged signals with disproportionate single-disease contributions, and these were interpreted conservatively rather than as fully shared effects. Together, these observations support the single common-factor model as a parsimonious and biologically coherent representation of shared CLD liability, while acknowledging that COPD aligns most closely with, and contributes most strongly to, the shared factor.

This interpretation is consistent with the direction of current respiratory genetics. Recent large-scale studies have shown that respiratory disease loci often span lung function, COPD, asthma, and fibrotic phenotypes, and that individual genetic effects can vary across ancestry, smoking history, age, cellular context, and clinical definition [11,15]. Bronchiectasis has historically received less genetic attention than asthma or COPD, but recent work has emphasized its links with epithelial integrity, host defense, infection susceptibility, and inflammatory regulation [9]. The mvCLD framework adds to this literature by asking whether part of this overlap can be modeled directly, rather than inferred indirectly from pairwise comparisons or post hoc overlap among single-disease GWAS results. This distinction matters because shared liability may be missed when genetic discovery is organized strictly around diagnostic labels that are clinically useful but biologically incomplete, particularly in respiratory medicine where overlap, misclassification, and comorbidity are common.

The biological signal emerging from mvCLD is most coherently understood as an epithelial–immune and cytokine-regulatory axis. A focused interpretation is more useful than a long gene-by-gene account: several established respiratory susceptibility regions and pathways converge, including epithelial alarmin signaling, IL-1/IL-18 receptor biology, type 2 cytokine signaling, and the 17q12-q21 asthma locus. The 17q12-q21 region, represented by genes such as ORMDL3 and GSDMB, remains one of the most reproducible asthma-associated regions and has been linked to gene regulation in airway and immune contexts [20]. In the present analysis, its strong mvCLD signal suggests that this locus may also contribute to a broader respiratory liability component, although the available data do not establish whether the same effector gene or cell state is relevant across all four diseases. Transcriptomic prioritization reinforced this interpretation but should not be read as definitive causal gene assignment. The TWAS/FOCUS results converged on several genes within the same major regions highlighted by the mvCLD GWAS, particularly 17q12-q21 and 2q12, and several prioritized genes also showed MAGMA Bonferroni-level support. This cross-method agreement strengthens gene-level prioritization in selected loci, but TWAS signals may still reflect local linkage disequilibrium, shared regulatory architecture, or correlated expression weights across nearby genes. This consideration is especially important in gene-dense regions such as 17q12-q21, where multiple nearby genes can show strong transcriptomic associations within the same association block.

The pathway-level findings further support a shared inflammatory architecture and argue against interpreting mvCLD as a purely generic marker of lung disease. The enrichment of interleukin, cytokine-receptor, JAK-STAT, and IL-4/IL-13 related terms is compatible with the growing therapeutic relevance of upstream and type 2 inflammatory pathways in respiratory medicine. TSLP blockade has shown clinical efficacy in severe uncontrolled asthma [21], and IL-4/IL-13 pathway inhibition has now shown benefit in COPD with evidence of type 2 inflammation [22]. Although these trials cannot validate mvCLD loci as treatment targets, they make the genetic convergence biologically plausible: shared inherited liability appears to concentrate in pathways already recognized as modulators of airway inflammation, epithelial activation, mucus biology, and exacerbation-prone disease biology. The more cautious implication is that multivariate genetics may help nominate pathway contexts for future stratified analyses, rather than immediately define drug indications across CLD diagnoses.

The mvCLD-specific lead variants deserve careful interpretation. Their value lies in the fact that they were not genome-wide significant in any of the four original univariate GWAS datasets yet became detectable in the multivariate analysis. This pattern supports the rationale of Genomic SEM: some variants may have modest, distributed effects across related phenotypes and therefore remain below threshold when each disease is analyzed separately. The additional fine-mapping support strengthens their credibility, particularly for variants that also showed high posterior probability or consensus evidence across methods. At the same time, mvCLD specificity defined by GWAS-by-subtraction is not equivalent to biological causality. These loci are best interpreted as prioritized mvCLD signals requiring replication, functional annotation, and disease-specific follow-up, especially because several map to noncoding or less well-characterized genomic regions. The disease-specific estimates now provided in Supplementary Table S10 and Supplementary Figure S1 underscore this caution: in the component datasets in which the effect allele was common enough for reliable estimation, the per-disease effects were close to the null, whereas in the FinnGen COPD and bronchiectasis datasets the effect-allele frequency was very low (<0.001), below the analysis minor-allele-frequency threshold, leaving the corresponding estimates imprecise. Accordingly, the “mvCLD-specific” label is operational rather than a claim of biological specificity or novelty, and external comparison and functional validation will be required to establish whether these signals are genuinely respiratory-relevant.

The spatial and heritability-partitioning analyses place these findings in a regulatory genomic context. The enrichment of conserved regions, promoters, enhancers, super-enhancers, open chromatin, and active histone marks is compatible with the established view that complex respiratory traits are shaped largely by regulatory rather than protein-altering variation. The gsMap analysis provided an anatomically plausible signal by showing the strongest overall enrichment in the lung annotation, consistent with the use of a spatial transcriptomic framework that maps GWAS-derived signals to tissue-resolved cellular contexts [23]. However, this result should not be overinterpreted. The atlas was derived from mouse embryonic tissue, and cross-species developmental spatial maps cannot substitute for adult human diseased lung tissue. Similarly, the broader cell-type and tissue enrichment analyses did not yield FDR-significant results, so nominal immune or T-cell related patterns should remain descriptive. The most balanced interpretation is that mvCLD signals show regulatory and spatial plausibility, but the precise adult human cell types and disease-stage contexts remain unresolved.

Several limitations should be considered. The input GWAS datasets were restricted to European ancestry and were selected for public availability, phenotype relevance, and compatibility with Genomic SEM, not because each represented the largest possible GWAS for that phenotype. Differences in phenotype ascertainment across UK Biobank, FinnGen, and GBMI-related data may introduce heterogeneity, and possible overlap among clinically related respiratory endpoints could influence genetic covariance estimates. Bronchiectasis had a comparatively small case number and low SNP heritability estimate, which may reduce its contribution to the latent factor. Conversely, COPD showed the highest factor loading and therefore aligned most closely with, and contributed most strongly to, the mvCLD factor. Although the positive and statistically significant loadings of all four phenotypes, the predominantly type 2 and epithelial–immune biology of the prioritized signals, and the variant-level Q_SNP diagnostic together argue against mvCLD representing a COPD- or smoking-specific construct, we did not perform a formal leave-one-trait-out re-estimation of the latent factor; the relative contribution of individual phenotypes to the shared signal should therefore be interpreted accordingly. The GWAS-by-subtraction approach depends on the power of the original univariate GWAS datasets and may classify some signals as mvCLD-specific because single-disease analyses were underpowered. Finally, fine-mapping, TWAS, FOCUS, pathway enrichment, and spatial mapping remain inferential tools. They prioritize variants, genes, pathways, and tissues but do not establish causality without independent replication and experimental validation. Relatedly, the pathway enrichment was not re-estimated after excluding highly pleiotropic or asthma-associated loci such as 17q12-q21, 2q12, the thymic stromal lymphopoietin region, and 5q31; although most of the enrichment-input genes lie outside these regions (Section 2.8), the enriched terms should be read as influenced by these well-characterized loci. More broadly, this study did not include functional validation, formal causal inference, clinical prediction modeling, drug-target validation, or treatment-response analysis. The prioritized loci and genes should therefore be regarded as candidate genes and candidate pathway contexts rather than as validated therapeutic targets, and the single-cell spatial result should be regarded as exploratory and hypothesis-generating pending validation in adult human lung single-cell or spatial datasets.

Two related features of the input data warrant more detailed consideration. First, the four GWAS datasets were generated under different ascertainment frameworks: asthma was defined by self-reported physician diagnosis in UK Biobank, COPD and bronchiectasis were register-based ICD-coded FinnGen endpoints that shared a common control group, and IPF was a multi-biobank meta-analysis assembled through the GBMI. Such differences in case definition, control selection, and the age, comorbidity, and diagnostic-coding structure of each cohort can influence the genetic covariance matrix in two opposing directions: overlapping or shared samples, such as the common FinnGen control set used for COPD and bronchiectasis, can inflate apparent genetic covariance, whereas phenotypic misclassification, most plausibly for self-reported asthma, and heterogeneous case ascertainment can attenuate genetic correlations toward the null. Several features of the analysis limit these effects. Genetic covariances were estimated by cross-trait LDSC, whose bivariate intercept explicitly absorbs confounding from sample overlap and shared controls, so that the genetic correlation estimates are protected from inflation due to overlapping samples; the univariate LDSC intercepts were close to one (0.96 to 1.11; Supplementary Table S1), and the genome-wide genomic-control metrics for the assembled mvCLD GWAS did not indicate substantial systematic inflation (Section 2.4). Genomic SEM further weights each phenotype by the precision of its genetic (co)variance estimates, and the variant-level Q_SNP statistic flags signals whose cross-phenotype behavior is inconsistent with a shared factor. These procedures reduce but cannot fully eliminate ascertainment-driven heterogeneity, and the genetic covariance estimates and mvCLD associations should be interpreted with this caveat. Second, bronchiectasis was the least powered phenotype, with the smallest case count (2932) and the lowest SNP-heritability estimate (h^2^ = 0.003); although this estimate remained nominally non-zero (approximately 2.6 times its standard error), its three pairwise genetic correlations carried wider standard errors than the other pairs and are therefore less precise. Two considerations mitigate the concern that this distorts the latent factor: the three bronchiectasis genetic correlations were nonetheless all positive and nominally significant (with asthma, COPD, and IPF), and Genomic SEM down-weights imprecise, low-power traits through the sampling covariance matrix, so bronchiectasis contributes proportionately less to the shared factor and is unlikely to drive it, consistent with its intermediate rather than extreme factor loading. A modest case count by itself does not preclude an informative contribution: IPF is also a comparatively rare disease for which even the largest available GWAS meta-analyses include only several thousand to approximately eleven thousand cases, and it was represented here by 6257 cases yet showed the highest SNP-heritability of the four phenotypes, illustrating that case count and heritability are not interchangeable. The lower precision of the bronchiectasis estimates should nevertheless be borne in mind, and these results would benefit from confirmation as larger bronchiectasis GWAS become available. Finally, this study did not include an independent replication cohort, and no new data were incorporated, so formal replication was beyond its scope; the mvCLD factor and its downstream signals should therefore be regarded as requiring external confirmation. The most suitable strategy for such replication would be to apply the identical analytical pipeline, namely cross-trait LDSC followed by Genomic SEM common-factor modeling, to non-overlapping GWAS of the same four phenotypes drawn from independent biobanks and, where possible, additional ancestries, and to test whether the positive genetic correlations, the single common-factor structure, the factor loadings, and the mvCLD lead variants and prioritized genes are reproduced; this would ideally be complemented by out-of-sample polygenic validation, in which mvCLD-derived posterior effect sizes are applied to an independent cohort. Within the present data, several internal analyses already guard against non-reproducible signals: the variant-level Q_SNP heterogeneity statistic functions as a built-in sensitivity analysis that flags variants inconsistent with the shared factor, fine-mapping required concordance between two methods with different priors (SuSiE and FINEMAP), and the near-unity LDSC intercepts argue against substantial confounding. These checks reduce, but cannot replace, the need for independent replication, which remains an essential next step before the shared-architecture model and its prioritized signals are considered established.

## 4. Materials and Methods

### 4.1. Ethical Compliance

This study used publicly available summary-level GWAS statistics. All original GWAS datasets were generated by the corresponding study teams under their institutional ethics approvals and informed-consent procedures. No individual-level data or identifiable personal information were accessed or analyzed; therefore, no additional ethics approval was required.

### 4.2. Selection of Univariate Input GWAS Datasets

We included four phenotypes representing major clinical dimensions of CLD: asthma, COPD, bronchiectasis, and IPF. These phenotypes capture chronic airway inflammation, irreversible airflow limitation, structural airway damage, and fibrotic interstitial lung disease, respectively, and were therefore used to model the shared genetic architecture of CLD-related traits. All GWAS summary statistics were derived from publicly available large-scale datasets of European ancestry.

The asthma dataset was obtained from a GWAS of White British participants in UK Biobank, including 56,167 asthma cases and 352,255 controls, with asthma defined by self-reported physician diagnosis [24]. The COPD and bronchiectasis datasets were obtained from FinnGen Release 12 (https://r12.finngen.fi/, accessed on 19 May 2026) [25]; the input datasets used in this study included 24,138 COPD cases and 409,070 controls, and 2932 bronchiectasis cases and 409,070 controls. The IPF dataset was obtained from a GWAS meta-analysis related to the Global Biobank Meta-analysis Initiative and included 6257 IPF cases and 947,616 controls [26]. The four input datasets differed in their phenotype-ascertainment frameworks. Asthma cases were defined by self-reported physician-diagnosed asthma in UK Biobank, whereas the COPD and bronchiectasis endpoints (FinnGen codes J10_COPD and J10_BRONCHIECTASIS) were defined from nationwide Finnish health-register data using harmonized ICD-code-based disease criteria, and the two FinnGen endpoints were analyzed against a common control group of 409,070 individuals. The IPF dataset was assembled as a multi-biobank GWAS meta-analysis released through the Global Biobank Meta-analysis Initiative, in which harmonized phenotype definitions were applied across contributing biobanks. These datasets therefore differ in case definition (self-report, register-based ICD coding, and harmonized meta-analysis criteria), control selection, and the underlying age, comorbidity, and diagnostic-coding structure of each cohort; the potential influence of this heterogeneity on the genetic covariance matrix and on the mvCLD associations is considered in the Discussion. All phenotypes were restricted to participants of European ancestry to reduce the influence of population structure on subsequent genetic covariance estimation and multivariate GWAS analysis. Details of the univariate GWAS datasets are summarized in Table 1.

### 4.3. Quality Control of Univariate Input GWAS Summary Statistics

To construct the multivariate chronic lung disease (mvCLD) model, we performed standardized quality control (QC) and harmonization of the GWAS summary statistics for asthma, COPD, bronchiectasis, and IPF. All included GWAS datasets had undergone sample-level and variant-level QC in the original studies; in the present analysis, we further standardized the single-nucleotide polymorphisms (SNPs) used for linkage disequilibrium score regression (LDSC) and Genomic SEM.

The analysis was restricted to autosomal SNPs, and allele alignment was performed using the European ancestry reference panel from the 1000 Genomes Project Phase 3. SNPs with a reference-panel minor allele frequency < 0.01, missing or non-finite effect estimates or standard errors, invalid *p* values, or unsuccessful allele alignment with the reference panel were excluded. After QC and harmonization, the retained high-quality SNPs were used for LDSC, Genomic SEM, and mvCLD GWAS analyses. Because this minor-allele-frequency threshold was applied to the harmonized reference-panel allele frequency, variants that were common in the reference panel were retained even where their allele frequency was substantially lower in an individual input cohort; accordingly, the per-cohort effect-allele frequencies reported for some variants (for example, in the FinnGen COPD and bronchiectasis datasets in Supplementary Table S10) can fall below 0.01 even though every analyzed variant had a reference-panel minor allele frequency of at least 0.01. All analyses were performed in the GRCh37/hg19 genome build, and the original GWAS summary statistics were lifted over to GRCh37/hg19 where necessary before harmonization. Each input GWAS was imputed as described in its original publication (Table 1). Allele alignment and harmonization used the 1000 Genomes Project Phase 3 European-ancestry reference panel, and the same European-ancestry reference framework was used for the linkage-disequilibrium (LD) reference data across analyses: European LD scores derived from 1000 Genomes Phase 3 for LDSC and Genomic SEM, and a 1000 Genomes Phase 3 European LD reference for fine-mapping (echolocatoR), transcriptome-wide association and gene-level fine-mapping (FUSION and FOCUS), and PRS-CS.

### 4.4. Genomic Structural Equation Modeling

We applied Genomic SEM [19] using the GenomicSEM R package v.0.0.5 to integrate genetic signals across asthma, COPD, bronchiectasis, and IPF. Genomic SEM estimates the shared genetic architecture across correlated phenotypes using summary-level data and can account for potential sample overlap and differences in sample size across GWAS datasets. Specifically, the cross-trait LDSC bivariate intercept absorbs confounding arising from overlapping samples or shared controls, including the common control group shared by the two FinnGen endpoints, so that the resulting genetic covariance estimates are protected from inflation due to sample overlap, and Genomic SEM weights each phenotype by the precision of its genetic (co)variance estimates through the LDSC sampling covariance matrix.

In the first stage, cross-trait LDSC was used to estimate the empirical genetic covariance matrix and the corresponding sampling covariance matrix among the four CLD phenotypes. In the second stage, we fitted a single common-factor model to represent the shared genetic susceptibility across the four CLD phenotypes; this latent factor was defined as mvCLD. Model fit was assessed using the standardized root mean square residual (SRMR), model χ^2^ statistic, Akaike information criterion, and comparative fit index (CFI). A single common-factor model was selected because all four phenotypes were positively genetically correlated and because a one-factor structure is the most parsimonious representation of a shared liability across the four traits; with only four indicators, more complex multifactor solutions are weakly identified and not statistically justified by the observed genetic-correlation structure. Model adequacy was therefore evaluated both globally, using the model χ^2^ test, CFI, and SRMR, and at the variant level, using the SNP-level heterogeneity statistic (Q_SNP; Section 4.5), which functioned as a sensitivity analysis of the common-factor assumption.

After confirming acceptable model fit, autosomal SNP effects were incorporated into the Genomic SEM framework to estimate the association between each SNP and the mvCLD latent factor, generating SNP-level mvCLD GWAS results representing the shared genetic signal across the four input GWAS datasets.

### 4.5. SNP-Level Heterogeneity Testing in Genomic SEM

To evaluate whether individual variant effects in the mvCLD GWAS were adequately captured by the common latent factor, we examined the SNP-level heterogeneity statistic (Q_SNP) generated by Genomic SEM. The Q_SNP test evaluates whether the effects of a given SNP across the four CLD phenotypes show phenotype-specific differences beyond the mvCLD common factor. Thus, Q_SNP was used as an auxiliary diagnostic metric to distinguish broadly shared signals from potentially heterogeneous signals driven by specific CLD phenotypes. In this way, Q_SNP additionally served as a built-in sensitivity analysis of the common-factor model: variants whose cross-phenotype effects were poorly explained by the shared factor, including any signals disproportionately driven by a single phenotype such as COPD, were flagged as heterogeneous and interpreted conservatively rather than treated as fully shared mvCLD effects.

### 4.6. Multilevel and Genomic Control Evaluation of Genomic SEM Results

To evaluate the robustness of the SNP-level mvCLD GWAS results, we summarized mvCLD association signals under multiple significance thresholds, including the conventional genome-wide significance threshold (*p* < 5 × 10^−8^), a stringent threshold (*p* < 5 × 10^−12^), and a more stringent signal-stability threshold (*p* < 5 × 10^−16^).

We also performed genomic control assessment using two-step LDSC to evaluate potential genome-wide statistical inflation in the mvCLD GWAS. Before analysis, mvCLD GWAS summary statistics were harmonized with the HapMap3 SNP reference list and checked according to the standard LDSC QC procedure, including missingness, INFO score, minor allele frequency, *p*-value range, non-SNP variants, strand ambiguity, duplicated rsIDs, insufficient sample size, and allele mismatch. Harmonized summary statistics were then merged with European linkage disequilibrium (LD) score reference data, and two-step LDSC with cutoff = 30 was used to estimate the mean χ^2^ statistic, genomic control lambda (λGC), LDSC intercept, and observed-scale SNP heritability. All SNP-heritability and genetic-correlation estimates (Supplementary Tables S1 and S2) and the heritability of the mvCLD factor are reported on the observed scale; we did not convert them to the liability scale, so no population-prevalence assumptions were required. Observed-scale estimates were used consistently across the univariate inputs, the Genomic SEM common-factor model, and the stratified-LDSC heritability partitioning, ensuring internal comparability.

### 4.7. Genomic Locus Definition and mvCLD-Specific Lead Variant Identification

Functional Mapping and Annotation of Genome-Wide Association Studies (FUMA) [27] was used to define genomic loci, identify lead SNPs, and perform functional annotation for the mvCLD GWAS results. SNPs reaching genome-wide significance (*p* < 5 × 10^−8^) were entered into FUMA, and independent significant SNPs and lead SNPs were defined according to LD structure. Lead SNPs with low mutual LD (*r*^2^ < 0.100) were used to represent the primary association signals within mvCLD-associated genomic loci.

To distinguish signals newly detected through the mvCLD shared genetic architecture from signals already significant in the component disease GWASs, we used a variant-level GWAS-by-subtraction strategy. Specifically, genome-wide significant lead SNPs from the mvCLD GWAS were compared with the original univariate GWAS summary statistics for asthma, COPD, bronchiectasis, and IPF. A lead SNP was classified as an mvCLD-specific lead SNP if it reached genome-wide significance in the mvCLD GWAS but did not reach genome-wide significance in any of the four original univariate GWAS datasets (*p* ≥ 5 × 10^−8^). This definition indicates discovery within the present multivariate framework and should not be interpreted as proof that the variant has never been reported in any external GWAS or functional study. For transparency, the disease-specific effect estimates, standard errors, *p* values, effect-allele frequencies, and effect directions for the mvCLD-specific lead variants in each of the four component GWASs are reported in Supplementary Table S10 and visualized as forest plots in Supplementary Figure S1, allowing direct inspection of each variant’s behavior across the component phenotypes.

In addition, Multi-marker Analysis of GenoMic Annotation (MAGMA) [28], implemented in FUMA, was used for gene-based association analysis. MAGMA integrates association signals across multiple SNPs within a gene to estimate gene-level associations with the mvCLD latent factor.

### 4.8. Fine-Mapping

To further prioritize candidate functional variants within mvCLD-associated loci, we performed fine-mapping using Sum of Single Effects (SuSiE) and FINEMAP [29,30] through the echolocatoR R package v.2.0.3 [31]. For each lead SNP identified in the mvCLD GWAS, a genomic region extending 250 kb upstream and downstream was extracted, and posterior probabilities were estimated for all candidate variants within the region.

Credible sets were constructed using a 95% posterior probability threshold to identify candidate variants most likely to explain regional association signals. High-confidence candidate variants were defined as variants with individual posterior probability ≥ 0.950 in both SuSiE and FINEMAP. Consensus SNPs were defined as variants with concordant credible-set support across SuSiE and FINEMAP under the echolocatoR fine-mapping framework. The mean posterior probability across the two methods was also calculated as an auxiliary measure of integrated evidence strength. Fine-mapping was performed using an LD reference panel matched to the European ancestry of the input GWAS data, consistent with the 1000 Genomes Project Phase 3 European-ancestry reference used for harmonization (Section 4.3). Posterior probabilities were interpreted as conditional on this LD reference and on the single- and multiple-causal-variant models implemented in SuSiE and FINEMAP. Because credible-set composition and posterior probabilities can be sensitive to LD-reference mismatch, posterior probabilities approaching 1.000 were interpreted as reflecting very small (frequently single-variant) credible sets, and high-posterior variants were treated as prioritized candidates requiring replication in independent, ancestry-matched data and experimental validation rather than as confirmed causal variants.

### 4.9. Transcriptome-Wide Association Study

After defining mvCLD-associated genomic loci and completing variant-level fine mapping, we performed a transcriptome-wide association study (TWAS) to evaluate whether genetically predicted gene expression was associated with the broader mvCLD latent factor. This analysis was designed as gene-level prioritization of the overall mvCLD association architecture, rather than as a direct one-to-one mapping of only the mvCLD-specific lead variants. The analysis was conducted using the Functional Summary-based Imputation (FUSION) pipeline [32] with precomputed expression quantitative trait locus weight models from the Genotype-Tissue Expression (GTEx) v8 resource [33]. In addition to tissue-specific expression weights, GTEx v8 cross-tissue expression weights generated by sparse canonical correlation analysis (sCCA) were used. The sCCA1, sCCA2, and sCCA3 features represent the first three cross-tissue canonical expression components and should not be interpreted as individual tissue-specific panels.

A total of 37,917 available gene–tissue pairs were analyzed, and transcriptome-wide significance was defined using a Bonferroni-corrected threshold of *p* < 1.319 × 10^−6^. To distinguish transcriptomic association signals from accompanying signals attributable to LD, TWAS-significant regions were further analyzed using Fine-mapping Of CaUsal gene Sets (FOCUS) [34]. FOCUS estimates the posterior inclusion probability (PIP) of genes whose genetically regulated expression is associated with the phenotype and constructs credible sets of candidate causal genes. FUSION and FOCUS results were then integrated to prioritize candidate genes with both TWAS significance and high posterior support.

### 4.10. Gene-Set and Pathway Enrichment Analysis

To characterize the functional features reflected by mvCLD gene-level signals, we performed gene-set and pathway enrichment analysis. Genes associated with mvCLD in MAGMA gene-based analysis were used as input for functional annotation and enrichment testing through the GENE2FUNC module of FUMA. MAGMA-significant genes used for GENE2FUNC analysis were selected according to a Bonferroni-corrected *p* value < 0.05.

GENE2FUNC analysis covered multiple gene-set categories from the Molecular Signatures Database (MSigDB) [35], including canonical pathways, Gene Ontology (GO) biological processes, cellular components, molecular functions, cell-type signatures, immunologic signatures, and other functional annotation datasets. Enrichment analysis was performed using the default FUMA GENE2FUNC background gene set, and statistical significance was defined as FDR-corrected *p* value < 0.05.

### 4.11. Cell-Type and Tissue-Specific Enrichment Analysis

To evaluate whether mvCLD gene-level signals showed preferences for specific cell types, tissues, or epigenomic annotations, we performed cross-dataset enrichment analysis. The datasets included the Tabula Muris single-cell transcriptomic dataset, GTEx tissue expression data, multitissue transcriptomic annotations, and multitissue epigenomic annotations. Epigenomic annotations included chromatin marks such as H3K4me1, H3K27ac, H3K4me3, H3K36me3, H3K9ac, and DNase hypersensitivity sites.

For each cell-type, tissue, or epigenomic annotation, enrichment coefficients, standard errors, and corresponding *p* values were estimated for association with mvCLD gene-level signals, followed by FDR correction within each dataset. FDR-corrected *p* value < 0.05 was considered statistically significant. Results with nominal *p* value < 0.05 but without FDR significance were interpreted only as exploratory enrichment trends and not as definitive cell-type or tissue-specific evidence.

### 4.12. Single-Cell Resolution Spatial Enrichment Mapping

To characterize the spatial distribution of mvCLD genetic signals at cellular and anatomical resolution, we integrated mvCLD GWAS summary statistics with single-cell spatial transcriptomic data. We used genetic spatial mapping of complex traits (gsMap) [23] to project human GWAS-derived polygenic association signals onto a spatially resolved single-cell transcriptomic atlas.

A mouse embryo spatial transcriptomic atlas at embryonic day 16.5 (E16.5) was used for cross-species spatial enrichment analysis. For each spatial location, gsMap estimated the enrichment significance of mvCLD genetic signals, which was displayed as −log_10_(*p*) on the spatial map. Annotation-level overall enrichment was summarized using the median *p* value across all spatial locations within each annotation, whereas the maximum −log_10_(*p*) was used to describe the strongest local enrichment signal within that annotation.

### 4.13. Heritability Partitioning Across Genomic Regions

To estimate the relative contribution of different functional genomic regions to mvCLD SNP heritability, we performed stratified linkage disequilibrium score regression (S-LDSC) [36]. S-LDSC partitions genome-wide SNP heritability across predefined functional genomic annotations and evaluates whether each annotation category shows heritability enrichment relative to the proportion of SNPs it contains.

We used the baseline functional annotation model to evaluate the distribution of mvCLD heritability across genomic regions. The annotation categories included protein-coding regions, introns, untranslated regions, transcription start sites, promoters, enhancers, super-enhancers, evolutionarily conserved regions, DNase I hypersensitivity sites, transcription factor binding sites, and active chromatin marks such as H3K27ac, H3K4me1, H3K4me3, and H3K9ac. For each annotation, we estimated the proportion of SNPs, proportion of SNP heritability, enrichment, standard error, and corresponding *p* value, with FDR < 0.05 used as the multiple-testing-corrected significance threshold.

### 4.14. Summary Statistics-Based Polygenic Weight Aggregation

To describe the genome-wide cumulative distribution of mvCLD-associated polygenic effect-size weights, we derived posterior SNP effect sizes from GWAS summary statistics using Polygenic Risk Score with Continuous Shrinkage (PRS-CS) [37]. PRS-CS was applied within a Bayesian regression framework by integrating mvCLD GWAS summary statistics with an external LD reference panel. Posterior SNP effect sizes generated by PRS-CS were further aggregated by chromosome. For each autosome, we calculated the number of included SNPs and the signed sum of posterior SNP effect sizes as a descriptive measure of chromosome-level polygenic weight distribution. This signed sum was used purely as a descriptive aggregate and was not assigned a biological interpretation, because its magnitude and sign are influenced by the arbitrary effect-allele coding, LD structure, variant density, and cancellation between variants with opposing effect directions.

## 5. Conclusions

In conclusion, this study identifies a measurable shared genetic component across asthma, COPD, bronchiectasis, and IPF. The mvCLD framework captures cross-phenotype genetic susceptibility, identifies mvCLD-specific lead variants missed by single-disease GWASs, and prioritizes epithelial–immune, cytokine-regulatory, exploratory lung-enriched spatial, and regulatory genomic contexts. These findings support multivariate genetic modeling as a complementary strategy for discovering shared respiratory disease biology while preserving disease-specific clinical interpretation.

## Figures and Tables

**Table 1:** Characteristics of the univariate genome-wide association study (GWAS) summary statistics used in this study.

Phenotype	GWAS ID	Cases	Controls	Sample Size (N)	Ancestry
Asthma	GCST90014325	56,167	352,255	408,422	European
COPD	finngen_R12_J10_COPD	24,138	409,070	433,208	European
Bronchiectasis	finngen_R12_J10_BRONCHIECTASIS	2932	409,070	412,002	European
IPF	GCST90399720	6257	947,616	953,873	European

Abbreviations: GWAS, genome-wide association study; COPD, chronic obstructive pulmonary disease; IPF, idiopathic pulmonary fibrosis.

**Table 2:** mvCLD-specific lead variants identified by multivariate GWAS-by-subtraction and supported by fine-mapping.

SNP	Chr	Position	Nearest Gene/Region	Effect/Other Allele	Beta	SE	mvCLD *p* Value	Fine-Mapping Status	Posterior Support
rs72736978	1	194599046	RP11-489C13.1	G/A	−0.047	0.006	6.52 × 10^−16^	High-confidence + consensus	SuSiE PP = 1.000; FINEMAP PP = 1.000
rs71208329	2	151915828	AC023469.1	C/T	0.048	0.005	1.95 × 10^−19^	High-confidence; not consensus	SuSiE PP = 1.000; FINEMAP PP = 1.000
rs11922517	3	125521965	RP11-379B18.6	T/C	−0.063	0.008	1.58 × 10^−16^	High-confidence + consensus	SuSiE PP = 1.000; FINEMAP PP = 1.000
rs4562243	7	90874487	*FZD1*	G/A	0.020	0.003	1.98 × 10^−11^	High-confidence + consensus	SuSiE PP = 1.000; FINEMAP PP = 1.000
rs118084239	14	88406143	*GALC*	T/C	0.072	0.010	2.43 × 10^−13^	High-confidence; not consensus	SuSiE PP = 1.000; FINEMAP PP = 1.000

Note: mvCLD-specific lead variants were defined as lead single-nucleotide polymorphisms (SNPs) that reached genome-wide significance in the multivariate chronic lung disease (mvCLD) genome-wide association study (GWAS), but did not reach genome-wide significance in any of the four component disease GWASs, including asthma, chronic obstructive pulmonary disease, bronchiectasis, and idiopathic pulmonary fibrosis. The effect allele is listed before the other allele. “High-confidence” indicates individual posterior probability (PP) ≥ 0.950 in both SuSiE and FINEMAP fine-mapping analyses. “Consensus” indicates concordant credible-set support across SuSiE and FINEMAP under the echolocatoR fine-mapping framework. Abbreviations: Chr, chromosome; FINEMAP, fine-mapping software package; PP, posterior probability; SE, standard error; SuSiE, Sum of Single Effects.

**Table 3:** Integrated transcriptomic and gene-based evidence for high-confidence TWAS/FOCUS-prioritized mvCLD candidate genes.

Gene	Chr	Regional mvCLD GWAS Lead/Index SNP	FOCUS-Supported Cross-Tissue sCCA Feature(s)	No. of Qualifying Associations	Minimum FOCUS-Supported TWAS *p* Value	TWAS Direction	Maximum FOCUS PIP	MAGMA Bonferroni Significance	Interpretive Context
*ORMDL3*	17	rs2305479	sCCA1	1	1.08 × 10^−51^	Positive	0.994	Yes	17q12-q21 asthma locus; epithelial/endoplasmic reticulum stress and airway immune regulation
*GSDMB*	17	rs2305479	sCCA2	1	3.89 × 10^−49^	Positive	1.000	Yes	17q12-q21 asthma locus; epithelial injury and inflammatory susceptibility
*TMEM182*	2	rs10197862	sCCA3	1	8.62 × 10^−40^	Positive	1.000	No	2q12-associated transcriptomic signal; interpret cautiously
*IL18RAP*	2	rs10197862	sCCA2	1	8.97 × 10^−33^	Positive	1.000	Yes	IL-18 receptor pathway; cytokine and host-defense signaling
*SMAD3*	15	rs17293632	sCCA1; sCCA2	2	3.75 × 10^−30^	Positive	1.000	Yes	TGF-β/remodeling and fibrosis-related regulatory biology
*WNT11*	11	rs2155219	sCCA3	1	6.40 × 10^−27^	Positive	1.000	No	Developmental/remodeling signal; requires further validation
*IL18R1*	2	rs10197862	sCCA1; sCCA3	2	8.23 × 10^−25^	Mixed	1.000	Yes	IL-18 receptor pathway; cytokine and host-defense signaling
*IL1R1*	2	rs10197862	sCCA2	1	2.15 × 10^−22^	Positive	1.000	Yes	IL-1 receptor pathway; epithelial–immune activation
*CCNI2*	5	rs848	sCCA2	1	2.30 × 10^−22^	Negative	1.000	Yes	5q31 cytokine-region signal; supportive transcriptomic evidence
*BACH2*	6	rs1847472	sCCA1	1	3.72 × 10^−19^	Negative	1.000	Yes	Immune-cell differentiation and lymphocyte regulatory biology
*IRF1*	5	rs1295686	sCCA3	1	1.30 × 10^−18^	Negative	1.000	Yes	Interferon and inflammatory transcriptional regulation
*SLC22A5*	5	rs848	sCCA3	1	9.80 × 10^−18^	Positive	0.997	Yes	5q31 region; carnitine transporter locus with immune–metabolic context
*D2HGDH*	2	rs7563672	sCCA1; sCCA3	2	7.80 × 10^−15^	Negative	1.000	Yes	2q37-associated transcriptomic signal; interpret cautiously
*SLC25A46*	5	rs1837253	sCCA3	1	2.88 × 10^−14^	Negative	1.000	No	TSLP/WDR36-region transcriptomic signal; mitochondrial membrane-related biology
*STAC3*	12	rs3024971	sCCA3	1	1.37 × 10^−9^	Negative	0.997	No	12q13-associated transcriptomic signal; interpret cautiously
*ZNF652*	17	rs12940887	sCCA1; sCCA3	2	1.76 × 10^−9^	Mixed	0.998	Yes	17q21-q22-associated transcriptomic signal with mixed direction across sCCA features
*CLEC16A*	16	rs12935657	sCCA1	1	2.25 × 10^−9^	Positive	0.964	Yes	16p13 immune-regulatory locus; autoimmune and antigen-processing context
*RAB5B*	12	rs705704	sCCA1	1	8.19 × 10^−9^	Positive	1.000	Yes	Vesicle trafficking/endosomal biology; MAGMA-supported gene-level signal
*UHRF2*	9	rs1888909	sCCA2; sCCA3	2	9.27 × 10^−9^	Mixed	1.000	No	9p24-associated transcriptomic signal with mixed direction across sCCA features
*NDFIP1*	5	rs7733850	sCCA1	1	4.03 × 10^−8^	Positive	0.999	Yes	Immune-regulatory/adaptor biology; MAGMA-supported gene-level signal
*KIAA2026*	9	rs3939286	sCCA2	1	9.22 × 10^−7^	Positive	0.967	No	9p24-associated transcriptomic signal; interpret cautiously

Note: The table summarizes 21 distinct genes derived from 26 high-confidence TWAS/FOCUS associations meeting both transcriptome-wide significance and FOCUS posterior inclusion probability (PIP) > 0.950. sCCA indicates sparse canonical correlation analysis. The sCCA1, sCCA2, and sCCA3 features represent cross-tissue canonical expression components derived from GTEx v8 expression weights rather than individual tissue-specific panels. The FOCUS-supported TWAS *p* value is the smallest TWAS *p* value among the qualifying FOCUS-supported associations for each gene. “Mixed” direction indicates that qualifying associations for the same gene showed opposite TWAS *Z*-score directions across supported sCCA features. Abbreviations: Chr, chromosome; FOCUS, Fine-mapping Of CaUsal gene Sets; GTEx, Genotype-Tissue Expression; GWAS, genome-wide association study; MAGMA, Multi-marker Analysis of GenoMic Annotation; mvCLD, multivariate chronic lung disease; PIP, posterior inclusion probability; sCCA, sparse canonical correlation analysis; TWAS, transcriptome-wide association study.

## Data Availability

The GWAS summary statistics used in this study are publicly available from the GWAS Catalog, FinnGen Release 12, and Global Biobank Meta-analysis Initiative-related resources, as described in Table 1. Derived mvCLD summary statistics and analysis scripts are available from the corresponding author upon reasonable request.

## Supplementary Materials

The following supporting information can be downloaded at https://www.mdpi.com/article/10.3390/ijms27156946/s1.

## Abbreviations

CFI, comparative fit index; CLD, chronic lung disease; COPD, chronic obstructive pulmonary disease; E16.5, embryonic day 16.5; FDR, false discovery rate; FINEMAP, fine-mapping software package; FOCUS, Fine-mapping Of CaUsal gene Sets; FUMA, Functional Mapping and Annotation of Genome-Wide Association Studies; FUSION, Functional Summary-based Imputation; GENE2FUNC, gene-based functional annotation module of FUMA; GO, Gene Ontology; gsMap, genetic spatial mapping of complex traits; GTEx, Genotype-Tissue Expression; GWAS, genome-wide association study; IPF, idiopathic pulmonary fibrosis; KEGG, Kyoto Encyclopedia of Genes and Genomes; LD, linkage disequilibrium; LDSC, linkage disequilibrium score regression; MAGMA, Multi-marker Analysis of GenoMic Annotation; MSigDB, Molecular Signatures Database; mvCLD, multivariate chronic lung disease; PIP, posterior inclusion probability; PP, posterior probability; PRS-CS, Polygenic Risk Score with Continuous Shrinkage; QC, quality control; Q_SNP, SNP-level heterogeneity statistic; sCCA, sparse canonical correlation analysis; S-LDSC, stratified linkage disequilibrium score regression; SE, standard error; SEM, structural equation modeling; SNP, single-nucleotide polymorphism; SRMR, standardized root mean square residual; SuSiE, Sum of Single Effects; TWAS, transcriptome-wide association study; λGC, genomic control lambda.
