# Supplementary material for: Integrative Multivariate Genomics Identifies Shared Epithelial–Immune and Cytokine-Regulatory Mechanisms Across Major Chronic Lung Diseases
Source: Int J Mol Sci. 2026 Aug 2;27(15):6946. doi: 10.3390/ijms27156946 (PMC13466783; doi:10.3390/ijms27156946)
Supplement: Supplementary file 1 [file ijms-27-06946-s001.zip › Supplementary Figure S1.pdf]

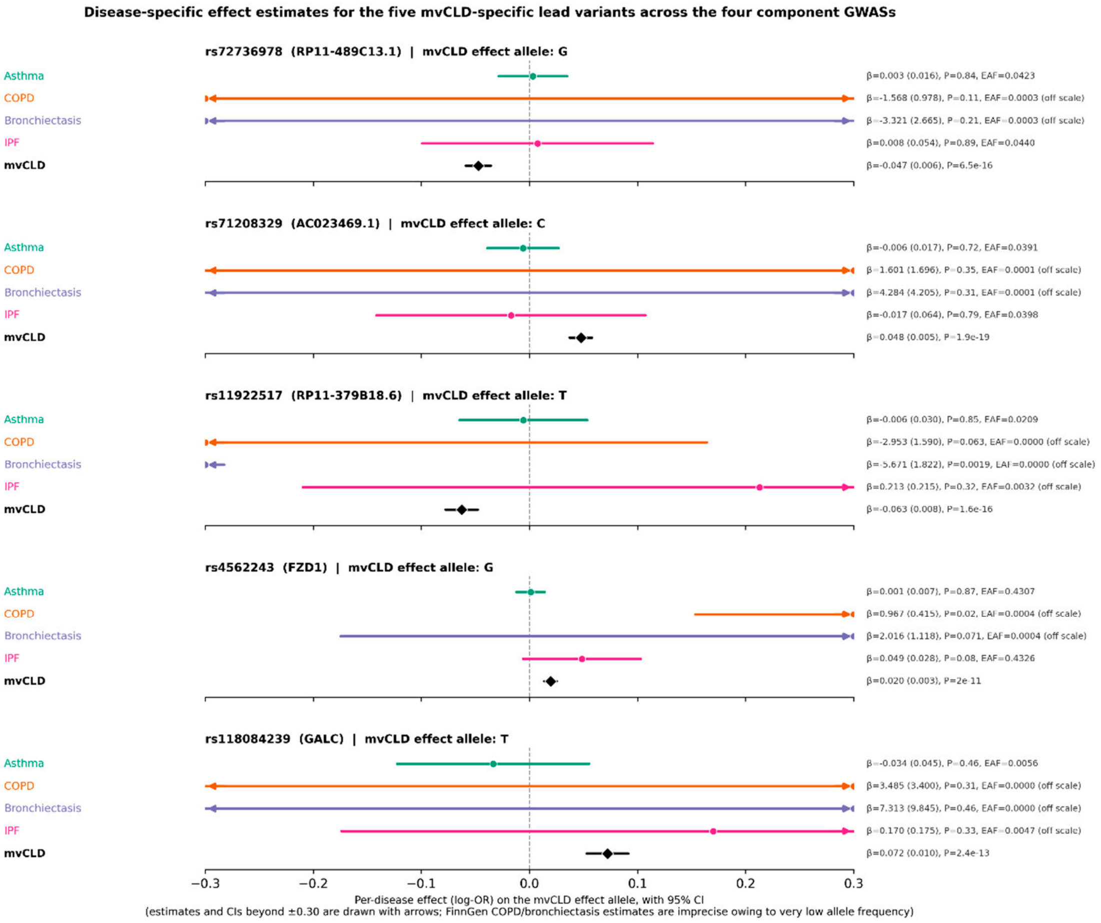

Supplementary Figure S1. Disease-specific effect estimates for the five mvCLD-specific lead variants across the four component GWASs. Forest plots show the per-variant effect estimate (log-odds ratio) with 95% confidence interval for rs72736978, rs71208329, rs11922517, rs4562243, and rs118084239 in asthma, COPD, bronchiectasis, and IPF, together with the corresponding mvCLD estimate. All effects are aligned to the mvCLD effect allele. Each row is annotated with the effect estimate, standard error, *P* value, and effect-allele frequency; estimates and confidence intervals extending beyond the plotted range are indicated with arrows. The COPD and bronchiectasis estimates are imprecise because the effect allele is very rare in the FinnGen datasets (effect-allele frequency < 0.001), below the minor-allele-frequency threshold used for the multivariate analysis. Abbreviations: CI, confidence interval; COPD, chronic obstructive pulmonary disease; EAF, effect-allele frequency; GWAS, genome-wide association study; IPF, idiopathic pulmonary fibrosis; mvCLD, multivariate chronic lung disease; OR, odds ratio; SE, standard error.
