# Supplementary material for: Integrative Multivariate Genomics Identifies Shared Epithelial–Immune and Cytokine-Regulatory Mechanisms Across Major Chronic Lung Diseases
Source: Int J Mol Sci. 2026 Aug 2;27(15):6946. doi: 10.3390/ijms27156946 (PMC13466783; doi:10.3390/ijms27156946)
Supplement: Supplementary file 1 [file ijms-27-06946-s001.zip › Supplementary_Tables.pdf]

Supplementary Tables

Supporting Information — Supplementary Tables S1–S23

Each supplementary table is presented below in a fixed, non-interactive format with a numbered caption, a legend, and a brief interpretation of the result. Large genome-scale result tables (Supplementary Tables 5, 6, 9–14, 16, and 19–21) are too large to be reproduced in full within the document; these are provided as separate plain-text comma-separated (.csv) data files, and their captions, legends, interpretations, and column schemas are given below.

List of Supplementary Tables

Table	Title
S1	Univariate LDSC estimates for the four input GWASs.
S2	Pairwise genetic correlation matrix for the four CLD phenotypes.
S3	Model-fit indices for the single common-factor Genomic SEM model.
S4	Parameter estimates for the mvCLD Genomic SEM common-factor model.
S5	Genome-wide significant SNP-level Genomic SEM associations for mvCLD (provided as a separate data file).
S6	Highly significant SNP-level Genomic SEM associations for mvCLD (provided as a separate data file).
S7	FUMA-defined genomic loci and lead SNPs for mvCLD.
S8	MAGMA gene-based association results for mvCLD.
S9	Functional annotation of mvCLD lead SNPs (provided as a separate data file).
S10	GWAS-by-subtraction classification of mvCLD-specific lead SNPs (provided as a separate data file).
S11	Fine-mapping output for mvCLD loci using SuSiE and FINEMAP (provided as a separate data file).
S12	FUSION transcriptome-wide association study results for mvCLD (provided as a separate data file).
S13	FOCUS fine-mapping results for mvCLD TWAS signals (provided as a separate data file).
S14	Integrated TWAS and FOCUS prioritization table for mvCLD (provided as a separate data file).
S15	MAGMA Bonferroni-significant genes used for pathway analysis.
S16	FUMA gene2func gene-set and pathway enrichment results for mvCLD (provided as a separate data file).
S17	Tabula Muris cell-type enrichment results for mvCLD-associated genes.
S18	GTEx tissue-level enrichment results for mvCLD-associated genes.
S19	Multi-tissue transcriptomic annotation enrichment results for mvCLD-associated genes (provided as a separate data file).
S20	Multi-tissue epigenomic annotation enrichment results for mvCLD-associated genes (provided as a separate data file).
S21	gsMap single-cell spatial enrichment results for mvCLD in the E16.5 mouse embryo atlas (provided as a separate data file).
S22	S-LDSC heritability enrichment across functional genomic annotations.
S23	Chromosome-level aggregation of PRS-CS posterior effect-size weights.

Supplementary Table S1. Univariate LDSC estimates for the four input GWASs.

The table summarizes univariate linkage disequilibrium score regression (LDSC) estimates for asthma, COPD, bronchiectasis, and IPF, including SNP heritability-related metrics, genomic control statistics, and intercept-related estimates. Phenotypes are ordered by descending SNP-based heritability.

Interpretation. All four input GWAS datasets yielded estimable SNP-based heritability (h^2 = 0.047, 0.038, 0.003, and 0.120 for asthma, COPD, bronchiectasis, and IPF, respectively), confirming a measurable additive genetic component for each phenotype and supporting their suitability as inputs to the multivariate model. The comparatively low heritability and small case number for bronchiectasis indicate that its contribution to the shared factor should be interpreted with caution.

Phenotype	NSNPs	h2_se	λGC	Mean_ChiSquare	Intercept_se	Ratio_se
IPF	952423	0.1202 (0.0261)	1.0402	1.0518	1.0009 (0.0081)	0.0172 (0.1569)
Asthma	1173732	0.0473 (0.0049)	1.171	1.3413	0.958 (0.0149)	0.123 (0.0436)
COPD	1159806	0.0383 (0.0031)	1.3687	1.4357	1.1086 (0.0097)	0.2493 (0.0222)
Bronchiectasis	1159791	0.0029 (0.0011)	1.0361	1.0353	1.0118 (0.0071)	0.3333 (0.2003)

Supplementary Table S2. Pairwise genetic correlation matrix for the four CLD phenotypes.

The table reports pairwise LDSC genetic correlation estimates among asthma, COPD, bronchiectasis, and IPF. Values summarize the shared genetic architecture used to construct the mvCLD common-factor model. Rows and columns follow the same phenotype order; the values form a symmetric genetic-correlation matrix.

Interpretation. The four CLD phenotypes were positively genetically correlated (the genetic correlation of asthma with COPD, bronchiectasis, and IPF was 0.387, 0.257, and 0.123, respectively), indicating shared additive genetic liability across clinically distinct lung diseases. This pervasive positive correlation provides the empirical basis for modelling a single shared genetic factor (mvCLD).

	**Asthma**	**COPD**	**Bronchiectasis**	**IPF**
Asthma	0.0473 (0.0049)	0.3867 (0.0392)	0.2566 (0.0945)	0.1228 (0.0606)
COPD	0.3867 (0.0392)	0.0383 (0.0031)	0.4357 (0.1002)	0.4394 (0.0768)
Bronchiectasis	0.2566 (0.0945)	0.4357 (0.1002)	0.0029 (0.0011)	0.5268 (0.1904)
IPF	0.1228 (0.0606)	0.4394 (0.0768)	0.5268 (0.1904)	0.1202 (0.0261)

Supplementary Table S3. Model-fit indices for the single common-factor Genomic SEM model.

The table reports model-fit statistics for the mvCLD common-factor model, including chi-square, degrees of freedom, AIC, CFI, and SRMR.

Interpretation. The single common-factor model showed acceptable fit (CFI = 0.985; SRMR = 0.096), indicating that one latent genetic factor adequately represents the shared genetic covariance among the four phenotypes and justifying its use as the mvCLD factor in all downstream analyses.

chisq	df	p_chisq	AIC	CFI	SRMR
5.26201901558936	2	0.0720057351847477	21.2620190155894	0.984619699396616	0.0962567756158785

Supplementary Table S4. Parameter estimates for the mvCLD Genomic SEM common-factor model.

The table provides unstandardized and standardized Genomic SEM estimates, standard errors, and P values for the mvCLD latent factor model. Parameters are grouped by type (factor loadings followed by (co)variance estimates).

Interpretation. All four phenotypes loaded positively on the latent factor (standardized loadings = 0.403, 0.940, 0.527, and 0.452 for asthma, COPD, bronchiectasis, and IPF), confirming that each disease contributes to mvCLD. COPD showed the strongest loading and asthma the weakest, indicating that the shared factor is informed by all four diseases but is not dominated by any single phenotype.

lhs	op	rhs	Unstandardized_Estimate	Unstandardized_SE	Standardized_Est	Standardized_SE	p_value
F1	=~	Asthma	0.113416915231931	0.0189712506090804	0.403134829628149	0.0674323662754629	2.25398885493807e-09
F1	=~	COPD	0.379992901030157	0.054934977943812	0.940103042323916	0.135909207622743	4.60854517012273e-12
F1	=~	Bronchiectasis	0.119706922226241	0.0245820704725658	0.526953526197006	0.108210977122335	1.11776696937737e-06
F1	=~	IPF	0.081791464001131	0.0149302647736915	0.45186767843002	0.0824842501117458	4.29595050103983e-08
Asthma	~~	Asthma	0.0662874840750551	0.00781714156900499	0.837482295306676	0.0987625966142156	2.25659014505061e-17
COPD	~~	COPD	0.0189855719098234	0.0404378821119464	0.116206290293378	0.247507458224378	0.638712527696758
Bronchiectasis	~~	Bronchiectasis	0.0372754925401869	0.0195081704263351	0.722320039482904	0.378026272278135	0.0560350294584666
IPF	~~	IPF	0.0260738608546537	0.00717158923217584	0.795815608994471	0.218888279422567	0.000277209732549917

Supplementary Table S5. Genome-wide significant SNP-level Genomic SEM associations for mvCLD.

The table lists SNP-level Genomic SEM associations reaching the genome-wide significance threshold for mvCLD ($P < 5 \times 10^{-8}$), including effect estimates and standard errors. Variants are ordered by ascending P value (most significant first).

Interpretation. A total of 2,067 variants reached genome-wide significance ($P < 5 \times 10^{-8}$) for mvCLD, showing that the multivariate factor captures a substantial and statistically robust set of shared association signals. These variants formed the primary input for locus definition, fine-mapping, and functional analyses.

Owing to its size (2,067 rows × 10 columns), the complete table is provided as a separate plain-text data file: "Supplementary Table 5 - Genome-wide significant SNP-level Genomic SEM associations for mvCLD.csv". The column headers are listed below for reference.

Columns:

SNP, CHR, BP, eaf, effect_allele, other_allele, beta, se, pval, N

Supplementary Table S6. Highly significant SNP-level Genomic SEM associations for mvCLD.

The table lists SNP-level Genomic SEM associations passing the more stringent threshold used to summarize high-confidence mvCLD association signals ($P < 5 \times 10^{-12}$). Variants are ordered by ascending P value (most significant first).

Interpretation. 1,409 variants surpassed the stringent threshold $P < 5 \times 10^{-12}$ and 916 surpassed $P < 5 \times 10^{-16}$, indicating that a large core of mvCLD associations is highly stable rather than driven by signals lying near the conventional significance boundary.

Owing to its size (1,409 rows × 10 columns), the complete table is provided as a separate plain-text data file: “Supplementary Table 6 - Highly significant SNP-level Genomic SEM associations for mvCLD.csv”. The column headers are listed below for reference.

Columns:

SNP, CHR, BP, eaf, effect_allele, other_allele, beta, se, pval, N

Supplementary Table *S7.* FUMA-defined genomic loci and lead SNPs for mvCLD.

The table summarizes independent genomic loci and lead SNPs identified from the mvCLD GWAS using FUMA, including genomic position, lead variant information, and mapped loci. Loci are ordered by ascending lead-SNP P value.

Interpretation. The genome-wide significant signal resolved into 30 independent genomic loci (177 independent significant SNPs and 53 lead SNPs), showing that mvCLD associations are distributed across many regions of the genome — a pattern consistent with a polygenic shared architecture rather than a few isolated major loci.

GenomicLocus	uniqID	rsID	chr	pos	p	start	end	nSNPs	nGWASSNPs	nIndSigSNPs	IndSigSNPs	nLeadSNPs	LeadSNPs
28	17:38062217:C:T	rs2305479	17	38062217	1.16990323799e-51	37741879	38219005	554	331	22	rs2305479;rs7213668;rs1031459;rs12946393;rs35557848;rs4065985;rs8069202;rs7212944;rs4580194;rs8077456;rs2302776;rs8078692;rs4795389;rs3794712;rs876493;rs407307;rs3809717;rs117097909;rs62066853;rs2227315;rs62065216;rs3087852	3	rs2305479;rs117097909;rs2227315
17	9:6213148:A:G	rs928412	9	6213148	2.90753254768e-50	5888194	6535500	422	244	16	rs10975416;rs343492;rs440366;rs10975357;rs62556407;rs76962799;rs4361812;rs117134689;rs72699188;rs928412;rs11791561;rs2169287;rs2169282;rs47	3	rs10975416;rs76962799;rs928412

GenomicLocus	uniqID	rsID	chr	pos	p	start	end	nSNPs	nGWASSNPs	nIndSigSNPs	IndSigSNPs	nLeadSNPs	LeadSNPs
											42211;rs369756;rs10124250		
4	2:102966549:A:G	rs10197862	2	102966549	3.54821971938e-49	102698360	103277862	494	347	23	rs10197862;rs11688559;rs2041739;rs4851005;rs13018263;rs1468788;rs112505882;rs66817110;rs9308860;rs72828033;rs11883987;rs1558641;rs10167431;rs9646944;rs12474199;rs72823611;rs12470864;rs1041973;rs1921622;rs10208293;rs56166614;rs1007027;rs141508079	2	rs10197862;rs56166614
10	5:110401872:C:T	rs1837253	5	110401872	3.20267333956e-43	110140757	110479610	251	159	7	rs7735519;rs1837253;rs1898671;rs2289278;rs6594499;rs58743367;rs12518884	3	rs7735519;rs1837253;rs6594499
18	10:9058671:A:G	rs1444782	10	9058671	4.55767731026e-43	8841669	9215062	345	223	21	rs11255938;rs10905467;rs72782676;rs11256016;rs2589561;rs827618;rs11256006;rs12356492;rs1444782;rs24	7	rs11255938;rs72782676;rs11256016;rs1444782;rs77000868;rs72784452;rs1149915

GenomicLocus	uniqID	rsID	chr	pos	p	start	end	nSNPs	nGWASSNPs	nIndSigSNPs	IndSigSNPs	nLeadSNPs	LeadSNPs
											88421;rs2797285;rs34289477;rs1149923;rs827620;rs144536148;rs7898135;rs77000868;rs118077070;rs72784452;rs72782677;rs1149915		
25	15:67448363:A:G	rs56375023	15	67448363	1.34527378547e-36	67371244	67674317	181	133	9	rs12592283;rs56375023;rs2289261;rs28360855;rs72743459;rs7166081;rs12912010;rs1470002;rs893475	3	rs12592283;rs56375023;rs7166081
6	2:242698640:A:G	rs34290285	2	242698640	2.73127169261e-35	242674268	242743943	126	78	9	rs34290285;rs4443030;rs6756901;rs11552660;rs7599470;rs62193063;rs741776;rs80241045;rs34077392	1	rs34290285
19	11:76293726:G:T	rs7936312	11	76293726	4.96471448273e-33	76057946	76312943	92	71	5	rs7936312;rs10899232;rs10899234;rs55646091;rs141420268	2	rs7936312;rs55646091
11	5:131996500:A:C	rs848	5	131996500	1.73174839669e-25	130423559	132138129	677	456	15	rs10477741;rs6894249;rs2522059;rs3749833;rs13164856;rs61352	4	rs10477741;rs736801;rs848;rs809125

GenomicLocus	uniqID	rsID	chr	pos	p	start	end	nSNPs	nGWASSNPs	nIndSigSNPs	IndSigSNPs	nLeadSNPs	LeadSNPs
											298;rs156109;rs200838;rs736801;rs743564;rs848;rs2243204;rs115008099;rs809125;rs256866		
26	16:11213021:A:T	rs35441874	16	11213021	2.85008015634e-22	11031741	11285678	221	139	5	rs35441874;rs11644510;rs12921922;rs3862469;rs7186106	1	rs35441874
21	12:57493727:G:T	rs3024971	12	57493727	1.47447308269e-20	57306224	57535266	80	54	8	rs3024971;rs35140931;rs11172067;rs111873928;rs3122929;rs11172110;rs703817;rs17547610	3	rs3024971;rs3122929;rs17547610
13	6:90984035:C:T	rs943689	6	90984035	5.88949618598e-20	90808352	91019304	128	95	5	rs943689;rs1394220;rs12212535;rs207274;rs72928038	1	rs943689
5	2:151915828:C:T	rs71208329	2	151915828	1.94825861611e-19	151915828	151926494	2	1	1	rs71208329	1	rs71208329
16	8:81291645:A:G	rs4739738	8	81291645	4.0829655081e-18	81246659	81329123	159	97	3	rs4739738;rs7000782;rs529240	1	rs4739738
8	3:125521965:C:T	rs11922517	3	125521965	1.5820085854e-16	125521965	125521965	1	1	1	rs11922517	1	rs11922517
1	1:152179152:C:T	rs12123821	1	152179152	1.94752757698e-16	151765695	152536650	15	12	4	rs12123821;rs12128071;rs115045402;rs61816766	2	rs12123821;rs61816766
2	1:194599046:A:G	rs72736978	1	194599046	6.5213652904e-16	194535326	194709891	35	1	1	rs72736978	1	rs72736978

GenomicLocus	uniqID	rsID	chr	pos	p	start	end	nSNPs	nGWASSNPs	nIndSigSNPs	IndSigSNPs	nLeadSNPs	LeadSNPs
30	19:33726578:A:C	rs117710327	19	33726578	1.386660116e-15	33721455	33726578	4	4	2	rs117710327;rs11672303	1	rs117710327
23	14:88406143:C:T	rs118084239	14	88406143	2.42701106326e-13	88406143	88406143	1	1	1	rs118084239	1	rs118084239
29	17:47439302:A:G	rs112502960	17	47439302	2.90929541605e-13	47303456	47481374	123	68	5	rs112502960;rs7209400;rs2671654;rs4987082;rs12601665	1	rs112502960
24	15:61069988:C:T	rs11071559	15	61069988	9.9378985501e-13	61032054	61070954	43	35	1	rs11071559	1	rs11071559
3	2:8443803:A:G	rs10178845	2	8443803	9.55957301176e-12	8438693	8468210	29	21	1	rs10178845	1	rs10178845
15	7:90874487:A:G	rs4562243	7	90874487	1.97916890998e-11	90867933	90894459	8	1	1	rs4562243	1	rs4562243
9	4:38798648:A:C	rs5743618	4	38798648	2.34739835772e-11	38763359	38894380	53	31	1	rs5743618	1	rs5743618
27	16:27349168:C:T	rs3785356	16	27349168	2.12323483161e-10	27302729	27372494	106	66	4	rs3785356;rs9940480;rs3024619;rs3024664	2	rs3785356;rs3024664
20	12:56396768:C:G	rs1689510	12	56396768	5.90282664889e-10	56368708	56482180	59	37	1	rs1689510	1	rs1689510
22	13:100032346:A:G	rs912131	13	100032346	7.18794705401e-10	99781378	100067446	173	113	2	rs912131;rs7982347	1	rs912131
7	3:33044339:C:T	rs9828592	3	33044339	4.00292984591e-09	33042712	33047662	6	6	1	rs9828592	1	rs9828592
12	5:141533062:A:G	rs449454	5	141533062	2.90442324969e-08	141435466	141543989	86	46	1	rs449454	1	rs449454
14	7:28172586:C:T	rs917115	7	28172586	4.87749156166e-08	28154215	28255530	20	15	1	rs917115	1	rs917115

Supplementary Table S8. MAGMA gene-based association results for mvCLD.

The table reports FUMA/MAGMA gene-based association results derived from mvCLD GWAS summary statistics and used for gene-level prioritization. Genes are ordered by ascending P value.

Interpretation. Gene-based testing implicated 136 genes (FDR < 0.05), with the strongest signals concentrated in epithelial alarmin and cytokine-receptor genes (e.g., IL1RL1, IL18RAP, TSLP, IL33, SMAD3, STAT6, and GSDMB). This early convergence on epithelial-immune genes indicates that mvCLD reflects coherent inflammatory biology rather than diffuse, non-specific signal.

GENE	CHR	START	STOP	NSNPS	NPARAM	N	ZSTAT	P	SYMBOL	FDR
ENSG00000170777	9	6328349	6331900	8	4	778535	8.2924	5.5511e-17	TPD52L3	1.020680757e-12
ENSG00000161395	17	37827375	37853050	51	4	778535	8.0023	6.1062e-16	PGAP3	4.76321364e-12
ENSG00000145777	5	110405760	110413722	10	4	778535	7.9726	7.7716e-16	TSLP	4.76321364e-12
ENSG00000169194	5	131991955	131996802	7	2	778535	7.9347	1.0547e-15	IL13	4.848192225e-12
ENSG00000166888	12	57489191	57525922	50	12	778535	7.7543	4.4409e-15	STAT6	1.44595368e-11
ENSG00000172057	17	38077294	38083854	9	4	778535	7.7466	4.7184e-15	ORMDL3	1.44595368e-11
ENSG00000113525	5	131877136	131892530	12	3	778535	7.7234	5.6621e-15	IL5	1.48727189571429e-11
ENSG00000115602	2	102927962	102968497	131	12	778535	7.6975	6.9389e-15	IL1RL1	1.520825544e-11
ENSG00000134987	5	110427414	110466200	88	13	778535	7.6867	7.5495e-15	WDR36	1.520825544e-11
ENSG00000115607	2	103035149	103069025	110	12	778535	7.675	8.2712e-15	IL18RAP	1.520825544e-11
ENSG00000141736	17	37844167	37886679	37	9	778535	7.6142	1.3267e-14	ERBB2	2.21763935454545e-11
ENSG00000180902	2	242673994	242708231	113	18	778535	7.5209	2.72e-14	D2HGDH	4.16772e-11
ENSG00000166949	15	67356101	67487533	298	48	778535	7.4954	3.3054e-14	SMAD3	4.67510690769231e-11
ENSG00000072682	5	131527531	131631008	195	20	778535	7.3479	1.0064e-13	P4HA2	1.32176262857143e-10
ENSG00000008838	17	38175350	38217468	73	9	778535	7.1679	3.8081e-13	MED24	4.66796898e-10
ENSG00000073605	17	38060848	38076107	33	4	778535	7.0453	9.2498e-13	GSDMB	1.06297545375e-09

GENE	CHR	START	STOP	NSNPS	NPARAM	N	ZSTAT	P	SYMBOL	FDR
ENSG00000103591	15	67493371	67547533	81	7	778535	7.0321	1.0169e-12	AAGAB	1.09986707647059e-09
ENSG00000115594	2	102681004	102796334	281	25	778535	7.008	1.2086e-12	IL1R1	1.2345849e-09
ENSG00000141744	17	37824234	37826728	4	2	778535	6.9679	1.608e-12	PNMT	1.55612084210526e-09
ENSG00000131435	5	131593364	131609147	36	6	778535	6.7135	9.5008e-12	PDLIM4	8.73456048e-09
ENSG00000115604	2	102927989	103015218	272	16	778535	6.6691	1.287e-11	IL18R1	1.12686042857143e-08
ENSG00000112182	6	90636248	91006627	645	74	778535	6.5686	2.5398e-11	BACH2	2.12269557272727e-08
ENSG00000038532	16	11038345	11276046	513	25	778535	6.3704	9.4267e-11	CLEC16A	7.53603186521739e-08
ENSG00000204913	17	38097727	38101000	6	2	778535	6.2614	1.9082e-10	LRRC3C	1.461919725e-07
ENSG00000131771	17	37782993	37792879	17	4	778535	6.2278	2.3647e-10	PPP1R1B	1.739189556e-07
ENSG00000197536	5	131746328	131811736	151	16	778535	6.1094	5e-10	C5orf56	2.96564516129032e-07
ENSG00000113522	5	131891711	131980313	89	6	778535	6.1094	5e-10	RAD50	2.96564516129032e-07
ENSG00000137033	9	6215805	6257983	107	16	778535	6.1094	5e-10	IL33	2.96564516129032e-07
ENSG00000141741	17	37884749	37887040	2	1	778535	6.1094	5e-10	MIEN1	2.96564516129032e-07
ENSG00000186075	17	38024417	38034149	26	3	778535	6.1094	5e-10	ZPBP2	2.96564516129032e-07
ENSG00000167914	17	38119226	38134019	37	7	778535	6.1094	5e-10	GSDMA	2.96564516129032e-07
ENSG00000198740	17	47366568	47439835	119	13	778535	6.075	6.1975e-10	ZNF652	3.48572945454545e-07
ENSG00000108344	17	38137050	38154213	46	5	778535	6.0735	6.256e-10	PSMD3	3.48572945454545e-07
ENSG00000103599	15	67547138	67794598	325	23	778535	5.9074	1.7375e-09	IQCH	9.39629779411765e-07
ENSG00000197375	5	131705444	131731306	72	8	778535	5.8335	2.7137e-09	SLC22A5	1.42562291142857e-06
ENSG00000170426	12	57316938	57328189	27	5	778535	5.7924	3.4686e-09	SDR9C7	1.77158745e-06

GENE	CHR	START	STOP	NSNPS	NPARAM	N	ZSTAT	P	SYMBOL	FDR
ENSG00000126351	17	38214543	38250120	44	13	778535	5.7808	3.7184e-09	THRA	1.84784380540541e-06
ENSG00000164402	5	132086509	132142933	73	10	778535	5.7258	5.1473e-09	SEPT8	2.49061592368421e-06
ENSG00000077238	16	27324989	27376099	133	18	778535	5.5672	1.2946e-08	IL4R	6.10354107692308e-06
ENSG00000197208	5	131630136	131679899	94	10	778535	5.4206	2.9693e-08	SLC22A4	1.3649129775e-05
ENSG00000131507	5	141488070	141534005	132	16	778535	5.3508	4.3779e-08	NDFIP1	1.96332798292683e-05
ENSG00000259202	15	67435072	67439277	8	4	778535	5.32	5.1885e-08	RP11-342M21.2	2.27145117857143e-05
ENSG00000154252	2	242716240	242743623	63	12	778535	5.2778	6.5372e-08	GAL3ST2	2.7953371255814e-05
ENSG00000139531	12	56390964	56400425	9	5	778535	5.27	6.8202e-08	SUOX	2.85006857727273e-05
ENSG00000108342	17	38171614	38174066	9	3	778535	5.263	7.0862e-08	CSF3	2.89542132e-05
ENSG00000131437	5	132028320	132073330	57	5	778535	5.2431	7.8948e-08	KIF3A	3.15568886086957e-05
ENSG00000134882	13	99853028	100038688	379	28	778535	5.2061	9.6442e-08	UBAC2	3.77293415744681e-05
ENSG00000069667	15	60780483	61521518	1651	199	778535	5.0995	1.7025e-07	RORA	6.5216390625e-05
ENSG00000113520	5	132009678	132018368	11	2	778535	5.0908	1.7825e-07	IL4	6.68257128e-05
ENSG00000125347	5	131817301	131826490	31	2	778535	5.0872	1.8172e-07	IRF1	6.68257128e-05
ENSG00000101204	20	61975420	62009753	151	32	778535	4.8948	4.9191e-07	CHRNA4	0.000177348022941176
ENSG00000164400	5	131409483	131411859	5	2	778535	4.8853	5.1642e-07	CSF2	0.000179405081320755
ENSG00000262039	17	47448102	47554350	234	46	778535	4.885	5.1713e-07	RP11-81K2.1	0.000179405081320755
ENSG00000135074	5	156822542	157002783	527	29	778535	4.8639	5.7544e-07	ADAM19	0.00019593732
ENSG00000156218	15	84322838	84708594	795	39	778535	4.8377	6.5677e-07	ADAMTSL3	0.000219564181636364
ENSG00000111540	12	56367697	56388490	33	5	778535	4.8065	7.6813e-07	RAB5B	0.000252207255535714

GENE	CHR	START	STOP	NSNPS	NPARAM	N	ZSTAT	P	SYMBOL	FDR
ENSG00000161405	17	37921198	38020441	195	6	778535	4.7862	8.4972e-07	IKZF3	0.000274101783157895
ENSG00000142599	1	8412457	8877702	527	26	778535	4.75	1.0173e-06	RERE	0.000322501639655172
ENSG00000245888	16	27279526	27301813	26	7	778535	4.6727	1.4862e-06	CTD-3203P2.2	0.000463165413559322
ENSG00000100599	14	92980118	93155339	499	88	778535	4.6595	1.5848e-06	RIN3	0.00048566196
ENSG00000158636	11	76155967	76264069	169	22	778535	4.6512	1.6501e-06	C11orf30	0.000497383421311476
ENSG00000109471	4	123372625	123377880	5	2	778535	4.6295	1.8327e-06	IL2	0.000536433428571429
ENSG00000173757	17	40351186	40428725	73	11	778535	4.6289	1.838e-06	STAT5B	0.000536433428571429
ENSG00000115598	2	102803433	102856462	167	13	778535	4.6251	1.8721e-06	IL1RL2	0.0005378484796875
ENSG00000205089	5	132083137	132089856	3	1	778535	4.5889	2.2284e-06	CCNI2	0.000630362935384615
ENSG00000164113	4	123300121	123350957	81	13	778535	4.5338	2.8963e-06	ADAD1	0.00080688285
ENSG00000169189	16	27236312	27280115	63	8	778535	4.5257	3.0096e-06	NSMCE1	0.000820384676470588
ENSG00000126561	17	40439565	40463961	42	7	778535	4.524	3.034e-06	STAT5A	0.000820384676470588
ENSG00000138688	4	123073488	123283913	264	21	778535	4.5013	3.3764e-06	KIAA1109	0.0008997372
ENSG00000126368	17	38249040	38256978	14	5	778535	4.4695	3.9201e-06	NR1D1	0.00102969826714286
ENSG00000131748	17	37793318	37819737	49	5	778535	4.4012	5.3839e-06	STARD3	0.00139427844084507
ENSG00000101246	20	62329996	62339377	8	3	778535	4.3467	6.9114e-06	ARFRP1	0.001764998775
ENSG00000139547	12	57345219	57353158	12	4	778535	4.3309	7.4261e-06	RDH16	0.00187046165342466
ENSG00000026036	20	62290653	62330037	131	17	778535	4.26	1.0223e-05	RTEL1-TNFRSF6B	0.0025401392027027
ENSG00000144730	3	57124010	57204334	164	20	778535	4.218	1.2322e-05	IL17RD	0.00302086152
ENSG00000065361	12	56473641	56497289	27	8	778535	4.2085	1.2855e-05	ERBB3	0.00311006427631579

GENE	CHR	START	STOP	NSNPS	NPARAM	N	ZSTAT	P	SYMBOL	FDR
ENSG00000146555	7	3341080	4308632	2762	113	778535	4.2019	1.3236e-05	SDK1	0.00316065366233766
ENSG00000236699	4	106473777	106629250	334	32	778535	4.1916	1.3848e-05	ARHGEF38	0.00326439969230769
ENSG00000197114	20	62338817	62367494	56	6	778535	4.1785	1.4672e-05	ZGPAT	0.00341486156962025
ENSG00000146197	6	35182190	35220856	48	12	778535	4.1749	1.4905e-05	SCUBE3	0.0034257279375
ENSG00000273154	20	62340216	62370456	62	6	778535	4.1695	1.5263e-05	RP4-583P15.15	0.003464701
ENSG00000152495	5	110559351	110830584	543	37	778535	4.1228	1.8719e-05	CAMK4	0.00419739332926829
ENSG00000117971	15	78916461	79020096	194	26	778535	4.1147	1.9387e-05	CHRNB4	0.00429480444578313
ENSG00000243509	20	62328021	62330037	6	2	778535	4.1007	2.0592e-05	TNFRSF6B	0.00450744171428571
ENSG00000203896	20	62366815	62370456	8	2	778535	4.0948	2.1128e-05	LIME1	0.00457035924705882
ENSG00000258366	20	62289163	62328416	128	16	778535	4.0896	2.161e-05	RTEL1	0.00462026825581395
ENSG00000180251	2	103089762	103150431	187	15	778535	4.0746	2.3051e-05	SLC9A4	0.00487170962068966
ENSG00000020633	1	25226002	25291612	107	26	778535	4.0456	2.6096e-05	RUNX3	0.00545258127272727
ENSG00000145012	3	187871072	188608460	1712	201	778535	4.0289	2.8023e-05	LPP	0.00578942585393259
ENSG00000136381	15	78729773	78793798	103	12	778535	4.0068	3.0778e-05	IREB2	0.0062879454
ENSG00000273047	20	62369623	62371751	5	2	778535	3.9948	3.2379e-05	RP4-583P15.14	0.00654233706593407
ENSG00000133103	13	40229764	40365802	290	17	778535	3.9669	3.6414e-05	COG6	0.00727765454347826
ENSG00000065029	6	35226686	35263762	60	9	778535	3.9333	4.189e-05	ZNF76	0.00828205838709678
ENSG00000188266	15	78799906	78829714	72	7	778535	3.8611	5.6427e-05	HYKK	0.0110374813723404
ENSG00000041357	15	78832747	78841604	24	5	778535	3.8576	5.725e-05	PSMA4	0.0110805868421053
ENSG00000125520	20	62371214	62374858	7	3	778535	3.8479	5.9579e-05	SLC2A4RG	0.0113872397010309

GENE	CHR	START	STOP	NSNPS	NPARAM	N	ZSTAT	P	SYMBOL	FDR
ENSG00000130584	20	62375019	62462597	306	18	778535	3.8458	6.0073e-05	ZBTB46	0.0113872397010309
ENSG00000169508	13	99946784	99959659	24	9	778535	3.7968	7.3295e-05	GPR183	0.0137517873979592
ENSG00000105855	7	20370325	20455377	266	17	778535	3.7595	8.5139e-05	ITGB8	0.0158126342727273
ENSG00000108306	17	37415384	37558776	213	12	778535	3.735	9.3857e-05	FBXL20	0.01725748659
ENSG00000169684	15	78857862	78887611	46	8	778535	3.7289	9.6142e-05	CHRNA5	0.0175026035049505
ENSG00000138061	2	38294116	38337044	115	19	778535	3.7257	9.7377e-05	CYP1B1	0.0175536362647059
ENSG00000177565	3	176737143	176915261	388	37	778535	3.7122	0.00010274	TBL1XR1	0.0183405862135922
ENSG00000174004	3	196366557	196388875	92	28	778535	3.6933	0.00011069	NRROS	0.0195697791346154
ENSG00000167258	17	37617764	37721160	147	11	778535	3.6792	0.00011697	CDK12	0.020483118
ENSG00000174125	4	38792298	38858438	207	18	778535	3.6606	0.00012581	TLR1	0.0218232874528302
ENSG00000138785	4	106603784	106817143	386	14	778535	3.6576	0.00012728	INTS12	0.0218719379439252
ENSG00000080644	15	78885394	78913637	63	8	778535	3.6513	0.00013048	CHRNA3	0.02221422
ENSG00000023892	6	35265595	35289548	34	7	778535	3.6431	0.00013469	DEF6	0.022524075
ENSG00000132478	17	73780681	73821886	86	15	778535	3.643	0.00013475	UNK	0.022524075
ENSG00000088387	13	99445741	99738879	749	35	778535	3.6383	0.00013724	DOCK9	0.0227336205405405
ENSG00000155666	16	27214807	27233089	25	7	778535	3.6323	0.00014044	KDM8	0.0230559846428571
ENSG00000137872	15	47476298	48066420	1413	62	778535	3.6114	0.0001523	SEMA6D	0.0247817707964602
ENSG00000268797	19	41307202	41404187	309	40	778535	3.6041	0.0001566	CTC-490E21.12	0.0252345724137931
ENSG00000136378	15	79051545	79103773	143	13	778535	3.601	0.00015852	ADAMTS7	0.0252345724137931
ENSG00000119408	9	127019885	127115586	204	25	778535	3.5999	0.0001592	NEK6	0.0252345724137931

GENE	CHR	START	STOP	NSNPS	NPARAM	N	ZSTAT	P	SYMBOL	FDR
ENSG00000138780	4	106629935	106768885	264	11	778535	3.5868	0.00016735	GSTCD	0.0262996961538462
ENSG00000149485	11	61567099	61596790	30	5	778535	3.5805	0.00017149	FADS1	0.0267219205932203
ENSG00000184361	17	43331760	43339479	18	5	778535	3.5372	0.00020217	SPATA32	0.0312378133613445
ENSG00000140403	15	78556428	78574538	44	10	778535	3.5286	0.00020887	DNAJA4	0.03200410575
ENSG00000174844	3	57327727	57530071	537	20	778535	3.5127	0.00022176	DNAH12	0.0336983563636364
ENSG00000167085	17	47481414	47492246	23	8	778535	3.5067	0.00022687	PHB	0.0341922843442623
ENSG00000100711	14	104182067	104200005	35	9	778535	3.4907	0.00024089	ZFYVE21	0.0360101173170732
ENSG00000135541	6	135604670	135818914	275	26	778535	3.4879	0.00024346	AHI1	0.0361007985483871
ENSG00000130203	19	45409011	45412650	4	2	778535	3.4831	0.00024785	APOE	0.0364577436
ENSG00000167081	9	128509624	128729656	212	21	778535	3.4495	0.00028079	PBX3	0.0409752835714286
ENSG00000132394	3	127872297	128127485	386	19	778535	3.4412	0.00028958	EEFSEC	0.0419252555905512
ENSG00000140600	15	84115980	84287495	335	52	778535	3.433	0.00029847	SH3GL3	0.0426621160465116
ENSG00000104941	19	46298968	46318577	44	10	778535	3.4322	0.00029931	RSPH6A	0.0426621160465116
ENSG00000163681	3	57741177	57914895	208	23	778535	3.4252	0.0003072	SLMAP	0.0434498953846154
ENSG00000099365	16	31000577	31021949	27	6	778535	3.4106	0.00032411	STX1B	0.0452947029545455
ENSG00000125245	13	99906968	99913998	13	6	778535	3.4097	0.00032517	GPR18	0.0452947029545455
ENSG00000129158	11	17809595	18034709	351	38	778535	3.4054	0.00033034	SERGEF	0.0454282394776119
ENSG00000103496	16	31044210	31054296	14	4	778535	3.4048	0.00033107	STX4	0.0454282394776119
ENSG00000112893	5	109025067	109205326	499	30	778535	3.3981	0.00033928	MAN2A1	0.046209936
ENSG00000170608	19	46367247	46377055	18	7	778535	3.3766	0.0003669	FOXA3	0.0496043404411765

Supplementary Table S9. Functional annotation of mvCLD lead SNPs.

The table summarizes functional annotations of mvCLD lead SNPs, including genomic context and variant-level annotation information from FUMA. Lead SNPs are ordered by ascending P value.

Interpretation. The 53 lead SNPs were located predominantly in noncoding regions (mainly intergenic and intronic), consistent with the view that complex respiratory traits are driven largely by regulatory rather than protein-altering variation. The strongest lead SNP, rs2305479 at 17q12 near GSDMB, marks the well-established 17q12–q21 respiratory locus.

Owing to its size (53 rows × 23 columns), the complete table is provided as a separate plain-text data file: “Supplementary Table 9 - Functional annotation of mvCLD lead SNPs.csv”. The column headers are listed below for reference.

Columns:

Novel, rsID, GenomicLocus, CHR, BP, nIndSigSNPs, IndSigSNPs, non_effect_allele, effect_allele, MAF, p, beta, se, nearestGene, dist, func, CADD, RDB, minChrState, commonChrState, Q, Q_df, Q_pval

Supplementary Table S10. GWAS-by-subtraction classification of mvCLD-specific lead SNPs.

The table compares mvCLD lead SNPs against the four input univariate GWASs and classifies variants as novel mvCLD-specific lead SNPs when they do not reach genome-wide significance in any input phenotype. Lead SNPs are ordered by ascending P value.

Interpretation. Five lead variants (rs72736978, rs71208329, rs11922517, rs4562243, and rs118084239) reached genome-wide significance for mvCLD but not for any individual disease GWAS, demonstrating the discovery value of the multivariate approach: variants with modest effects distributed across related phenotypes can become detectable when shared liability is modelled jointly. Here, mvCLD specificity denotes detection within this framework and not biological causality.

Owing to its size (53 rows × 54 columns), the complete table is provided as a separate plain-text data file: "Supplementary Table 10 - GWAS-by-subtraction classification of mvCLD-specific lead SNPs.csv". The column headers are listed below for reference.

Columns:

Novel, GenomicLocus, rsID, CHR, BP, nearestGene, non_effect_allele, effect_allele, MAF, beta, se, p, SNP_Asthma, pval_Asthma, CHR_Asthma, BP_Asthma, effect_allele_Asthma, other_allele_Asthma, eaf_Asthma, beta_Asthma, se_Asthma, N_Asthma, SNP_COPD, CHR_COPD, BP_COPD, effect_allele_COPD, other_allele_COPD, eaf_COPD, beta_COPD, se_COPD, pval_COPD, N_COPD, SNP_Bronchiectasis, CHR_Bronchiectasis, BP_Bronchiectasis, effect_allele_Bronchiectasis, other_allele_Bronchiectasis, eaf_Bronchiectasis, beta_Bronchiectasis, se_Bronchiectasis, pval_Bronchiectasis, N_Bronchiectasis, SNP_IPF, CHR_IPF, BP_IPF, effect_allele_IPF, other_allele_IPF, beta_IPF, se_IPF, eaf_IPF, pval_IPF, ncontrol_IPF, ncase_IPF, N_IPF

Supplementary Table S11. Fine-mapping output for mvCLD loci using SuSiE and FINEMAP.

The table contains the complete fine-mapping output used to prioritize candidate causal variants across mvCLD-associated loci, including posterior-probability estimates from SuSiE and FINEMAP and lead/consensus-SNP annotations. The high-confidence and consensus variant counts reported in the main text were obtained by applying the posterior-probability and lead/consensus-SNP criteria described in the Methods. Variants are ordered by descending mean posterior probability (SuSiE and FINEMAP), then by ascending P value.

Interpretation. Integrating SuSiE and FINEMAP, 18 variants met the high-confidence posterior-probability threshold (≥ 0.950 in both methods) and 12 were further supported as consensus variants, providing statistically prioritized candidate causal variants within mvCLD loci. Notably, all five mvCLD-specific lead variants were recovered in this high-confidence set, strengthening their credibility.

Owing to its size (43,463 rows × 24 columns), the complete table is provided as a separate plain-text data file: "Supplementary Table 11 - Fine-mapping output for mvCLD loci using SuSiE and FINEMAP.csv". The column headers are listed below for reference.

Columns:

NearestGene, SNP, CHR, POS, MAF, A1, A2, Effect, StdErr, P, N, tstat, leadSNP, SUSIE.CS, SUSIE.PP, FINEMAP.CS, FINEMAP.PP, FINEMAP.k, FINEMAP.PP_snp, Support, Consensus_SNP, mean.PP, mean.CS, FINEMAP.PP_config

Supplementary Table S12. FUSION transcriptome-wide association study results for mvCLD.

The table reports the complete FUSION TWAS output across sparse canonical correlation analysis (sCCA)-derived cross-tissue expression features, including gene-expression weight associations, Z statistics, and P values. The transcriptome-wide significant subset discussed in the main text was defined using the Bonferroni-corrected threshold described in the Methods. Associations are ordered by ascending TWAS P value.

Interpretation. TWAS identified 92 transcriptome-wide significant gene-expression associations (55 distinct genes), with the strongest signals at 17q12–q21 (ORMDL3, RARA, ERBB2, GSDMB) and 2q12 (IL18RAP, IL18R1, IL1R1). This links the mvCLD GWAS signal to genetically regulated expression of epithelial-immune genes; however, TWAS associations may reflect local linkage disequilibrium and shared regulatory architecture rather than direct causal genes, particularly in gene-dense regions such as 17q12–q21.

Owing to its size (37,917 rows × 29 columns), the complete table is provided as a separate plain-text data file: "Supplementary Table 12 - FUSION transcriptome-wide association study results for mvCLD.csv". The column headers are listed below for reference.

Columns:

ID, PANEL, CHR, P0, P1, HSQ, BEST.GWAS.ID, BEST.GWAS.Z, EQTL.ID, EQTL.R2, EQTL.Z, EQTL.GWAS.Z, NSNP, NWGT, MODEL, MODELCV.R2, MODELCV.PV, TWAS.Z, TWAS.P, COLOC.PP0, COLOC.PP1, COLOC.PP2, COLOC.PP3, COLOC.PP4, chr, start_hg38, end_hg38, strand, ensembl

Supplementary Table S13. FOCUS fine-mapping results for mvCLD TWAS signals.

The table reports FOCUS gene-level fine-mapping output, including posterior inclusion probabilities used to prioritize candidate causal genes. NULL-model entries have been removed. Genes are ordered by descending posterior inclusion probability.

Interpretation. Gene-level fine-mapping prioritized 50 genes with PIP > 0.500 (34 with PIP > 0.950), helping to distinguish candidate causal genes from neighbouring genes whose transcriptomic signals arise through shared regulatory variation within the same locus.

Owing to its size (1,128 rows × 21 columns), the complete table is provided as a separate plain-text data file: “Supplementary Table 13 - FOCUS fine-mapping results for mvCLD TWAS signals.csv”. The column headers are listed below for reference.

Columns:

block, ens_gene_id, ens_tx_id, mol_name, tissue, ref_name, type, chrom, tx_start, tx_stop, block_genes, trait, inference_pop1, inter_z_pop1, cv.R2_pop1, cv.R2.pval_pop1, ldregion_pop1, twas_z_pop1, pips_pop1, in_cred_set_pop1, eqtlfile

Supplementary Table S14. Integrated TWAS and FOCUS prioritization table for mvCLD.

The table integrates FUSION TWAS and FOCUS fine-mapping information for mvCLD. The high-confidence candidate genes discussed in the main text are those satisfying both transcriptome-wide significance and high FOCUS posterior support, as defined in the Methods. Associations are ordered by ascending TWAS P value.

Interpretation. Requiring both transcriptome-wide significance and high FOCUS posterior support yielded 21 high-confidence candidate genes (e.g., ORMDL3, GSDMB, IL18RAP, SMAD3, IL18R1, IL1R1, BACH2, IRF1, and CLEC16A), representing the most strongly supported transcriptomic candidates for mvCLD. These remain prioritized signals that require experimental validation rather than confirmed effector genes.

Owing to its size (37,917 rows × 31 columns), the complete table is provided as a separate plain-text data file: "Supplementary Table 14 - Integrated TWAS and FOCUS prioritization table for mvCLD.csv". The column headers are listed below for reference.

Columns:

ensembl, PANEL, ID, CHR, P0, P1, HSQ, BEST.GWAS.ID, BEST.GWAS.Z, EQTL.ID, EQTL.R2, EQTL.Z, EQTL.GWAS.Z, NSNP, NWGT, MODEL, MODELCV.R2, MODELCV.PV, TWAS.Z, TWAS.P, COLOC.PP0, COLOC.PP1, COLOC.PP2, COLOC.PP3, COLOC.PP4, chr, start_hg38, end_hg38, strand, pip, in_cred_set

Supplementary Table S15. MAGMA Bonferroni-significant genes used for pathway analysis.

The table lists MAGMA genes reaching Bonferroni-corrected significance and used as the input gene set for downstream gene-set and pathway enrichment analyses. Genes are ordered by ascending P value.

Interpretation. Under the conservative Bonferroni threshold (P < 0.05), 65 genes remained significantly associated with mvCLD, including canonical type 2 inflammatory and epithelial-immune genes (IL1R1, IL1RL1, IL18R1, IL18RAP, TSLP, WDR36, IL5, IL13, IL4, STAT6, SMAD3, IL4R, GSDMB, ORMDL3, and CLEC16A). Their survival under stringent multiple-testing correction indicates that the shared signal is robust and biologically focused on cytokine and epithelial-immune pathways; this gene set was used as the input for the pathway enrichment analysis.

GENE	SYMBOL	CHR	START	STOP	ZSTAT	P	P_bon
ENSG00000170777	TPD52L3	9	6328349	6331900	8.2924	5.5511e-17	1.020680757e-12
ENSG00000161395	PGAP3	17	37827375	37853050	8.0023	6.1062e-16	1.122746994e-11
ENSG00000145777	TSLP	5	110405760	110413722	7.9726	7.7716e-16	1.428964092e-11
ENSG00000169194	IL13	5	131991955	131996802	7.9347	1.0547e-15	1.93927689e-11
ENSG00000166888	STAT6	12	57489191	57525922	7.7543	4.4409e-15	8.16548283e-11
ENSG00000172057	ORMDL3	17	38077294	38083854	7.7466	4.7184e-15	8.67572208e-11
ENSG00000113525	IL5	5	131877136	131892530	7.7234	5.6621e-15	1.041090327e-10
ENSG00000115602	IL1RL1	2	102927962	102968497	7.6975	6.9389e-15	1.275855543e-10
ENSG00000134987	WDR36	5	110427414	110466200	7.6867	7.5495e-15	1.388126565e-10
ENSG00000115607	IL18RAP	2	103035149	103069025	7.675	8.2712e-15	1.520825544e-10
ENSG00000141736	ERBB2	17	37844167	37886679	7.6142	1.3267e-14	2.43940329e-10
ENSG00000180902	D2HGDH	2	242673994	242708231	7.5209	2.72e-14	5.001264e-10
ENSG00000166949	SMAD3	15	67356101	67487533	7.4954	3.3054e-14	6.07763898e-10
ENSG00000072682	P4HA2	5	131527531	131631008	7.3479	1.0064e-13	1.85046768e-09
ENSG00000008838	MED24	17	38175350	38217468	7.1679	3.8081e-13	7.00195347e-09
ENSG00000073605	GSDMB	17	38060848	38076107	7.0453	9.2498e-13	1.700760726e-08
ENSG00000103591	AAGAB	15	67493371	67547533	7.0321	1.0169e-12	1.86977403e-08
ENSG00000115594	IL1R1	2	102681004	102796334	7.008	1.2086e-12	2.22225282e-08
ENSG00000141744	PNMT	17	37824234	37826728	6.9679	1.608e-12	2.9566296e-08
ENSG00000131435	PDLIM4	5	131593364	131609147	6.7135	9.5008e-12	1.746912096e-07
ENSG00000115604	IL18R1	2	102927989	103015218	6.6691	1.287e-11	2.3664069e-07
ENSG00000112182	BACH2	6	90636248	91006627	6.5686	2.5398e-11	4.66993026e-07
ENSG00000038532	CLEC16A	16	11038345	11276046	6.3704	9.4267e-11	1.733287329e-06

GENE	SYMBOL	CHR	START	STOP	ZSTAT	P	P_bon
ENSG00000204913	LRRC3C	17	38097727	38101000	6.2614	1.9082e-10	3.50860734e-06
ENSG00000131771	PPP1R1B	17	37782993	37792879	6.2278	2.3647e-10	4.34797389e-06
ENSG00000197536	C5orf56	5	131746328	131811736	6.1094	5e-10	9.1935e-06
ENSG00000113522	RAD50	5	131891711	131980313	6.1094	5e-10	9.1935e-06
ENSG00000137033	IL33	9	6215805	6257983	6.1094	5e-10	9.1935e-06
ENSG00000141741	MIEN1	17	37884749	37887040	6.1094	5e-10	9.1935e-06
ENSG00000186075	ZPBP2	17	38024417	38034149	6.1094	5e-10	9.1935e-06
ENSG00000167914	GSDMA	17	38119226	38134019	6.1094	5e-10	9.1935e-06
ENSG00000198740	ZNF652	17	47366568	47439835	6.075	6.1975e-10	1.139534325e-05
ENSG00000108344	PSMD3	17	38137050	38154213	6.0735	6.256e-10	1.15029072e-05
ENSG00000103599	IQCH	15	67547138	67794598	5.9074	1.7375e-09	3.19474125e-05
ENSG00000197375	SLC22A5	5	131705444	131731306	5.8335	2.7137e-09	4.98968019e-05
ENSG00000170426	SDR9C7	12	57316938	57328189	5.7924	3.4686e-09	6.37771482e-05
ENSG00000126351	THRA	17	38214543	38250120	5.7808	3.7184e-09	6.83702208e-05
ENSG00000164402	SEPT8	5	132086509	132142933	5.7258	5.1473e-09	9.46434051e-05
ENSG00000077238	IL4R	16	27324989	27376099	5.5672	1.2946e-08	0.000238038102
ENSG00000197208	SLC22A4	5	131630136	131679899	5.4206	2.9693e-08	0.000545965191
ENSG00000131507	NDFIP1	5	141488070	141534005	5.3508	4.3779e-08	0.000804964473
ENSG00000259202	RP11-342M21.2	15	67435072	67439277	5.32	5.1885e-08	0.000954009495
ENSG00000154252	GAL3ST2	2	242716240	242743623	5.2778	6.5372e-08	0.001201994964
ENSG00000139531	SUOX	12	56390964	56400425	5.27	6.8202e-08	0.001254030174
ENSG00000108342	CSF3	17	38171614	38174066	5.263	7.0862e-08	0.001302939594
ENSG00000131437	KIF3A	5	132028320	132073330	5.2431	7.8948e-08	0.001451616876
ENSG00000134882	UBAC2	13	99853028	100038688	5.2061	9.6442e-08	0.001773279054
ENSG00000069667	RORA	15	60780483	61521518	5.0995	1.7025e-07	0.00313038675
ENSG00000113520	IL4	5	132009678	132018368	5.0908	1.7825e-07	0.00327748275
ENSG00000125347	IRF1	5	131817301	131826490	5.0872	1.8172e-07	0.00334128564
ENSG00000101204	CHRNA4	20	61975420	62009753	4.8948	4.9191e-07	0.00904474917

GENE	SYMBOL	CHR	START	STOP	ZSTAT	P	P_bon
ENSG00000164400	CSF2	5	131409483	131411859	4.8853	5.1642e-07	0.00949541454
ENSG00000262039	RP11-81K2.1	17	47448102	47554350	4.885	5.1713e-07	0.00950846931
ENSG00000135074	ADAM19	5	156822542	157002783	4.8639	5.7544e-07	0.01058061528
ENSG00000156218	ADAMTSL3	15	84322838	84708594	4.8377	6.5677e-07	0.01207602999
ENSG00000111540	RAB5B	12	56367697	56388490	4.8065	7.6813e-07	0.01412360631
ENSG00000161405	IKZF3	17	37921198	38020441	4.7862	8.4972e-07	0.01562380164
ENSG00000142599	RERE	1	8412457	8877702	4.75	1.0173e-06	0.0187050951
ENSG00000245888	CTD-3203P2.2	16	27279526	27301813	4.6727	1.4862e-06	0.0273267594
ENSG00000100599	RIN3	14	92980118	93155339	4.6595	1.5848e-06	0.0291397176
ENSG00000158636	C11orf30	11	76155967	76264069	4.6512	1.6501e-06	0.0303403887
ENSG00000109471	IL2	4	123372625	123377880	4.6295	1.8327e-06	0.0336978549
ENSG00000173757	STAT5B	17	40351186	40428725	4.6289	1.838e-06	0.033795306
ENSG00000115598	IL1RL2	2	102803433	102856462	4.6251	1.8721e-06	0.0344223027
ENSG00000205089	CCNI2	5	132083137	132089856	4.5889	2.2284e-06	0.0409735908

Supplementary Table S16. FUMA gene2func gene-set and pathway enrichment results for mvCLD.

The table reports FUMA gene2func enrichment results for MAGMA-significant mvCLD genes, including pathway, Gene Ontology, GWAS Catalog, immunologic, and other enrichment categories. Gene sets are ordered by ascending FDR-corrected P value.

Interpretation. Enrichment analysis of the Bonferroni-significant genes yielded 545 FDR-significant gene sets that converged on interleukin and cytokine-receptor signalling, JAK-STAT signalling, IL-1/IL-18 family signalling, IL-4/IL-13 signalling, TSLP signalling, and lung-fibrosis and asthma pathways. This convergence indicates that the shared genetic liability is concentrated in inflammatory pathways already recognised as central to airway disease and as emerging therapeutic targets, rather than representing a generic lung-disease marker.

Owing to its size (545 rows × 8 columns), the complete table is provided as a separate plain-text data file: "Supplementary Table 16 - FUMA gene2func gene-set and pathway enrichment results for mvCLD.csv". The column headers are listed below for reference.

Columns:
Category, GeneSet, N_genes, N_overlap, p, adjP, genes, link

Supplementary Table S17. Tabula Muris cell-type enrichment results for mvCLD-associated genes.

The table reports Tabula Muris single-cell enrichment estimates for mvCLD gene-level signals across mouse organ-derived cell types. Annotations are ordered by ascending P value.

Interpretation. No cell type reached FDR significance; the leading nominal signals (e.g., liver natural killer cells, and limb-muscle and lung T cells) are therefore exploratory only and should be read as descriptive trends rather than evidence of specific cell-type enrichment.

Name	Coefficient	Coefficient_std_error	Coefficient_P_value	FDR
Liver_natural_killer_cell	7.31453826585545e-09	2.67689292380145e-09	0.00314303893760005	0.277605813001974
Limb_Muscle_T_cell	1.64020342908107e-08	6.68468051138928e-09	0.007070233373359	0.277605813001974
Marrow_immature_T_cell	8.26687618312309e-09	3.60363221450106e-09	0.0108941027070612	0.277605813001974
Skin_epidermal_cell	3.35642732682432e-09	1.48213520370604e-09	0.0117689511463708	0.277605813001974
Lung_T_cell	7.18772665705541e-09	3.33844434862245e-09	0.0156586804259152	0.277605813001974
Trachea_mesenchymal_cell	1.52287191844273e-09	7.19014878172963e-10	0.0170876276907794	0.277605813001974
Pancreas_pancreatic_PP_cell	1.18742600252788e-09	5.65593672756226e-10	0.0178893755654249	0.277605813001974
Bladder_bladder_cell	1.44483755771783e-09	6.99745424465506e-10	0.0194707471414222	0.277605813001974
Spleen_T_cell	7.19118008326655e-09	3.56783465304891e-09	0.0219230723991725	0.277605813001974
Marrow_B_cell	7.94345605179519e-09	4.07708442101228e-09	0.0256884757273466	0.277605813001974
Fat_T_cell	6.37618992460595e-09	3.29681020744387e-09	0.0265535995045366	0.277605813001974
Trachea_blood_cell	7.21092261322913e-09	3.81003499620377e-09	0.0292046620108321	0.279878010937141
Skin_basal_cell_of_epidermis	2.65045396808127e-09	1.45714587672629e-09	0.0344606562345869	0.29266628847622
Lung_stromal_cell	1.21495999339349e-09	6.73544424695884e-10	0.0356289394666703	0.29266628847622
Marrow_regulatory_T_cell	1.00996517637413e-08	5.80156250608183e-09	0.0408549121759543	0.313220993348983
Limb_Muscle_lymphocyte	6.56342928184814e-09	3.89074978035025e-09	0.0458082394201663	0.329246720832445
Thymus_DN1_thymic_pro-T_cell	7.88898963641385e-09	4.95508725306262e-09	0.0556812268958998	0.376667123119322
Heart_professional_antigen_presenting_cell	1.79430702256349e-09	1.20042329307823e-09	0.0674926441339642	0.420111710652018
Bladder_bladder_urothelial_cell	1.71571280248938e-09	1.15910771803302e-09	0.0694097608903335	0.420111710652018
Heart_fibroblast	1.02830200303967e-09	7.09162484103572e-10	0.0735260349962403	0.422774701228381
Mammary_Gland_stromal_cell	2.75732946169173e-09	1.9527667002022e-09	0.0789732555668778	0.432472590009093
Fat_unknown_cell_type	2.99033956125489e-09	2.2589512772089e-09	0.0927891602094386	0.485034246549338
Lung_B_cell	4.26412722336447e-09	3.35816534794762e-09	0.102081714119767	0.488672571040816
Limb_Muscle_mesenchymal_stem_cell	1.53749478829359e-09	1.21642197719687e-09	0.103124248025686	0.488672571040816
Heart_leukocyte	3.40735503337231e-09	2.82942633165677e-09	0.114245228324236	0.488672571040816
Marrow_immature_NK_T_cell	6.68655719444639e-09	5.6890052583686e-09	0.119927891206874	0.488672571040816
Marrow_immature_natural_killer_cell	7.71639735669185e-09	6.64085097521907e-09	0.12262603531549	0.488672571040816
Marrow_naive_B_cell	3.42353503225733e-09	3.05007625666794e-09	0.130837184342298	0.488672571040816
Fat_natural_killer_cell	6.53169562855296e-09	5.97862344850924e-09	0.137304876240307	0.488672571040816
Pancreas_pancreatic_stellate_cell	9.01902115158631e-10	8.29519181660792e-10	0.138461202880976	0.488672571040816
Marrow_pre-	3.57113763163918e-09	3.32985309891753e-09	0.141756507831492	0.488672571040816

Name	Coefficient	Coefficient_std_error	Coefficient_P_value	FDR
natural_killer_cell				
Spleen_B_cell	2.94699818817816e-09	2.76379983203733e-09	0.143147423516821	0.488672571040816
Fat_mesenchymal_stem_cell_of_adipose	1.09874207596417e-09	1.03116674048279e-09	0.143317425292151	0.488672571040816
Marrow_mature_natural_killer_cell	5.69687869720781e-09	5.37229244943246e-09	0.144477107959893	0.488672571040816
Fat_myeloid_cell	3.32665488001678e-09	3.22808084676117e-09	0.151379132360155	0.494592000259599
Fat_B_cell	2.64621573390827e-09	2.60645190246439e-09	0.154991924444534	0.494592000259599
Marrow_basophil	4.72702338331675e-09	4.87379192129696e-09	0.166051617039894	0.494592000259599
Lung_leukocyte	4.44774594377805e-09	4.59620980463146e-09	0.166597450581405	0.494592000259599
Skin_stem_cell_of_epidermis	1.22638784547353e-09	1.28030421142418e-09	0.16905965747463	0.494592000259599
Lung_epithelial_cell_of_lung	1.79161214314687e-09	1.91428660595002e-09	0.174658127817394	0.494592000259599
Kidney_leukocyte	3.13043042403277e-09	3.43354375199705e-09	0.180958072999534	0.494592000259599
Limb_Muscle_macrophage	3.16949058087928e-09	3.5280254925818e-09	0.184492778741717	0.494592000259599
Pancreas_pancreatic_A_cell	5.87073931374683e-10	6.54690972473843e-10	0.184934400097068	0.494592000259599
Spleen_macrophage	5.03962283191239e-09	5.73668805003217e-09	0.189838943050726	0.49616996479167
Limb_Muscle_skeletal_muscle_satellite_cell	1.02105862949249e-09	1.20017315317637e-09	0.197451500788193	0.504598279792048
Lung_monocyte	1.58517967143003e-09	1.99851836365047e-09	0.213837225933324	0.53459306483331
Lung_myeloid_cell	3.98106813989861e-09	5.14607922097824e-09	0.219580165449008	0.537270617587998
Brain_Non-Myeloid_Bergmann_glial_cell	5.37562790170635e-10	7.60335707700986e-10	0.239781013807554	0.568560033898076
Pancreas_leukocyte	1.76634911552592e-09	2.52673444742443e-09	0.242256014443528	0.568560033898076
Lung_natural_killer_cell	3.22763250700059e-09	4.83377707898132e-09	0.252154659771233	0.577410139816783
Thymus_immature_T_cell	1.29155470435434e-09	1.97029688915966e-09	0.2560688446144	0.577410139816783
Liver_B_cell	1.65222338385666e-09	2.62809795116353e-09	0.264780437738131	0.579282742622717
Brain_Myeloid_macrophage	1.97284521728055e-09	3.1718211075298e-09	0.266973785730469	0.579282742622717
Marrow_granulocyte	1.25533761932363e-09	2.26313193637632e-09	0.289553212719456	0.616641101161804
Pancreas_pancreatic_D_cell	2.69027106997902e-10	5.1902467782459e-10	0.302113319784344	0.63169148682181
Limb_Muscle_B_cell	1.49101519540877e-09	2.98078552295705e-09	0.308464025901148	0.633452910332715
Marrow_immature_B_cell	1.01750009323603e-09	2.1028429430214e-09	0.31423949175479	0.633991957049138
Mammary_Gland_luminal_epithelial_cell_of_mammary_gland	5.96429629795251e-10	1.29719043406533e-09	0.32283501923983	0.639956036466127
Mammary_Gland_endothelial_cell	9.66594942977233e-10	2.17435980299141e-09	0.328325270882622	0.639956036466127
Skin_keratinocyte_stem_cell	3.4909384793822e-10	8.16542603584139e-10	0.334497833943974	0.641120848392617
Marrow_precursor_B_cell	7.78676873269865e-10	2.01486921714273e-09	0.349576078372228	0.659036869062397
Heart_unknown_cell_type	3.47734357117707e-10	9.93362020530477e-10	0.363147571908879	0.673580173701953
Pancreas_type_B_pancreatic_cell	1.47964462697317e-10	5.08060080879236e-10	0.385436110318633	0.703573852168932

Name	Coefficient	Coefficient_std_error	Coefficient_P_value	FDR
Tongue_basal_cell_of_epidermis	2.15500099833105e-10	8.03124647540193e-10	0.394223777747083	0.70837085063929
Lung_ciliated_columnar_cell_of_tracheobronchial_tree	2.41006211867625e-10	1.37696973911885e-09	0.430529400410734	0.761705862265145
Kidney_macrophage	9.82300893277852e-11	1.30990717984571e-09	0.470111288638516	0.81913330596105
Brain_Non-Myeloid_neuron	-1.00040492118683e-11	5.28956876148222e-10	0.507544660955265	0.871158746415754
Marrow_hematopoietic_precursor_cell	-1.00870390634079e-10	1.20077279680621e-09	0.533473596943855	0.902197994831519
Brain_Non-Myeloid_astrocyte	-7.88974643578679e-11	7.26590299451619e-10	0.543234529112276	0.905390881853794
Mammary_Gland_basal_cell	-1.32202033644532e-10	9.96828276650538e-10	0.55275410104214	0.908096023140659
Kidney_kidney_collecting_duct_epithelial_cell	-1.93476801998062e-10	9.95078443743017e-10	0.577081854931737	0.933710451998046
Trachea_epithelial_cell	-1.96070272978094e-10	9.17783952247914e-10	0.584583935163994	0.933710451998046
Limb_Muscle_endothelial_cell	-3.20520294763443e-10	1.167106245829e-09	0.608199053089928	0.950466909086333
Thymus_leukocyte	-4.53859256944524e-10	1.60089113883942e-09	0.611604793672945	0.950466909086333
Skin_leukocyte	-5.58013810466595e-10	1.80907016707266e-09	0.621131315958242	0.952401351135972
Tongue_keratinocyte	-2.93552715985274e-10	7.96862867521344e-10	0.643707091483009	0.967754889855179
Lung_unknown_cell_type	-6.2286927099511e-10	1.38576169606739e-09	0.673456530109014	0.967754889855179
Marrow_common_lymphoid_progenitor	-5.07742053063207e-10	1.0959826847485e-09	0.678416589085847	0.967754889855179
Brain_Non-Myeloid_brain_pericyte	-2.98351231000842e-10	6.28594037082987e-10	0.682475572061096	0.967754889855179
Large_Intestine_enteroendocrine_cell	-3.61894588840991e-10	7.62126932042884e-10	0.682552406479119	0.967754889855179
Marrow_granulocytopoietic_cell	-1.06495026321682e-09	2.20404221676723e-09	0.685516216994538	0.967754889855179
Limb_Muscle_skeletal_muscle_satellite_stem_cell	-4.27935115386003e-10	8.62779716522265e-10	0.690051312766302	0.967754889855179
Marrow_late_pro-B_cell	-5.86008286117581e-10	1.11276988251889e-09	0.700771690022575	0.968712080384442
Heart_smooth_muscle_cell	-4.23866779151683e-10	7.61866213539208e-10	0.711015320160692	0.968712080384442
Heart_erythrocyte	-5.55282300854274e-10	9.35332394778982e-10	0.723634832074424	0.968712080384442
Brain_Myeloid_microglial_cell	-5.47714660917622e-10	8.89301523178662e-10	0.731017457986812	0.968712080384442
Large_Intestine_Brush_cell_of_epithelium_proper_of_large_intestine	-7.66298234381126e-10	1.22409861680912e-09	0.734345878346489	0.968712080384442
Pancreas_pancreatic_acinar_cell	-6.82982482916052e-10	1.05515417034421e-09	0.741275331076791	0.968712080384442
Large_Intestine_large_intestine_goblet_cell	-6.36483403162703e-10	8.97569157113939e-10	0.760874704136669	0.983152707592325
Trachea_endothelial_cell	-8.64958640407854e-10	1.11724941503467e-09	0.78058953999546	0.992988257539279
Heart_myofibroblast_cell	-5.56319650769438e-10	7.02617730190644e-10	0.785755925531082	0.992988257539279
Brain_Non-Myeloid_oligodendrocyte	-6.64928426464217e-10	7.12583404393604e-10	0.824621934953789	0.999441055307275
Heart_endocardial_cell	-9.77704036127429e-10	9.99116307401902e-10	0.836103456774773	0.999441055307275
Kidney_endothelial_cell	-1.18589143914396e-09	1.13665052563879e-09	0.851600191388542	0.999441055307275
Pancreas_endothelial_cell	-1.09061626698323e-09	1.04076014858895e-09	0.852658479522172	0.999441055307275
Brain_Non-	-7.35115020235643e-10	6.99797619617956e-10	0.853248506744648	0.999441055307275

Name	Coefficient	Coefficient_std_error	Coefficient_P_value	FDR
Myeloid_oligodendrocyte_precursor_cell				
Pancreas_pancreatic_ductal_cell	-9.20275563378018e-10	8.60364324331487e-10	0.857608122360218	0.999441055307275
Liver_endothelial_cell_of_hepatic_sinusoid	-1.10928426910934e-09	9.68632494228365e-10	0.873938195469664	0.999441055307275
Large_Intestine_epithelial_cell_of_large_intestine	-8.76595620672083e-10	7.41220029368418e-10	0.881523850393488	0.999441055307275
Lung_lung_endothelial_cell	-1.13870667269584e-09	9.57488494970954e-10	0.882832116994564	0.999441055307275
Marrow_macrophage	-1.17951192810528e-09	9.47870961429329e-10	0.893320180579392	0.999441055307275
Heart_cardiac_muscle_cell	-9.95736354874644e-10	6.64838484388141e-10	0.932895910385257	0.999441055307275
Lung_classical_monocyte	-2.11962399371848e-09	1.39164690137439e-09	0.936133748578645	0.999441055307275
Marrow_Slamf1-negative_multipotent_progenitor_cell	-1.56713783113187e-09	1.0237815440997e-09	0.937082500447608	0.999441055307275
Liver_Kupffer_cell	-2.25411637863426e-09	1.410472673094e-09	0.944992793294626	0.999441055307275
Marrow_megakaryocyte-erythroid_progenitor_cell	-1.87410261373088e-09	1.13579455940677e-09	0.950532276606848	0.999441055307275
Marrow_granulocyte_monocyte_progenitor_cell	-2.39689258350556e-09	1.32084353313997e-09	0.965212532519553	0.999441055307275
Brain_Non-Myeloid_endothelial_cell	-1.63242759232613e-09	8.31663210416226e-10	0.975168019482733	0.999441055307275
Large_Intestine_enterocyte_of_epithelium_of_large_intestine	-1.43725475403138e-09	7.15915730257712e-10	0.977655780529008	0.999441055307275
Heart_endothelial_cell	-2.09583721437915e-09	1.00168434451435e-09	0.981794738938763	0.999441055307275
Marrow_Slamf1-positive_multipotent_progenitor_cell	-2.45059790439901e-09	1.13440169332155e-09	0.984623565975375	0.999441055307275
Fat_endothelial_cell	-2.01881269579992e-09	9.10009649070322e-10	0.986737984399692	0.999441055307275
Marrow_monocyte	-3.00677223964005e-09	1.2741331385252e-09	0.990859014124024	0.999441055307275
Liver_hepatocyte	-3.12365718479428e-09	1.0995880964723e-09	0.997749635749982	0.999441055307275
Kidney_epithelial_cell_of_proximal_tubule	-2.92840530323931e-09	8.98547762280559e-10	0.999441055307275	0.999441055307275

Supplementary Table S18. GTEx tissue-level enrichment results for mvCLD-associated genes.

The table reports GTEx tissue-level enrichment estimates used to evaluate whether mvCLD gene-level signals show broad tissue specificity. Annotations are ordered by ascending P value.

Interpretation. No tissue reached FDR significance. Whole blood showed the leading nominal signal and lung did not reach even nominal significance, indicating that bulk-tissue expression data do not resolve a single mvCLD-relevant tissue and that these results should be interpreted cautiously.

Name	Coefficient	Coefficient_std_error	Coefficient_P_value	FDR
Whole_Blood	1.39293980198676e-09	7.15465181069079e-10	0.0257733086150765	0.66629850005727
Cervix_Endocervix	9.20881151061215e-10	5.46489261752923e-10	0.0459860348282466	0.66629850005727
Cells_EBV-transformed_lymphocytes	1.08355511554227e-09	6.80790331073109e-10	0.0557357818203982	0.66629850005727
Prostate	8.80254992144629e-10	5.72644838523847e-10	0.0621253024759129	0.66629850005727
Skin_Sun_Exposed_(Lower_leg)	1.13758339213204e-09	7.42929435245463e-10	0.0628583490620066	0.66629850005727
Lung	8.34057029079416e-10	5.94058017790001e-10	0.0801595320283774	0.708075866250668
Uterus	7.15832993149563e-10	5.50814478594161e-10	0.0968707629048394	0.728049859145082
Skin_Not_Sun_Exposed_(Suprapubic)	8.09271320044069e-10	6.59504268821732e-10	0.109894318361522	0.728049859145082
Spleen	8.1065632521838e-10	7.01648547836895e-10	0.123971616958249	0.730055077643021
Minor_Salivary_Gland	4.87818130800758e-10	4.51582033643425e-10	0.140017090957076	0.742090582072502
Esophagus_Muscularis	5.73377860836188e-10	6.02861913038207e-10	0.170778535360348	0.756356886698136
Bladder	4.10789947703745e-10	4.32759070421084e-10	0.171250615856182	0.756356886698136
Colon_Transverse	4.6748944884456e-10	5.22965320813186e-10	0.185682200056464	0.757012046384047
Adipose_Visceral_(Omentum)	4.72673917913753e-10	5.94946077836918e-10	0.213457457479383	0.773036217066795
Ovary	5.15268086384754e-10	7.24941767551994e-10	0.23861286275408	0.773036217066795
Esophagus_Gastroesophageal_Junction	3.73315799263515e-10	5.87323815682605e-10	0.262511490537211	0.773036217066795
Thyroid	3.12973732391658e-10	4.93754088173589e-10	0.263084267902941	0.773036217066795
Breast_Mammary_Tissue	3.22141393286285e-10	5.21039324187337e-10	0.268199692372572	0.773036217066795
Stomach	3.0047574292157e-10	5.080752429356e-10	0.277126191023945	0.773036217066795
Cells_Transformed_fibroblasts	1.7188436627893e-10	4.90533175019682e-10	0.3630180839765	0.899151315827683
Artery_Aorta	1.42754284427319e-10	5.02967539823957e-10	0.388272611245556	0.899151315827683
Testis	1.06686611456589e-10	4.16613051959788e-10	0.398944216339805	0.899151315827683
Kidney_Cortex	1.30701429546804e-10	5.49881380737573e-10	0.406060682566326	0.899151315827683
Small_Intestine_Terminal_Ileum	1.56632749739259e-10	6.66950105504572e-10	0.407162859997441	0.899151315827683
Cervix_Ectocervix	8.51313487819483e-11	4.75736403288987e-10	0.428989867950678	0.909458520055438
Colon_Sigmoid	5.8317925667282e-12	5.6488587365687e-10	0.495881456048681	0.995821857444388
Fallopian_Tube	-5.67204284371962e-11	4.58089131551643e-10	0.549270956972888	0.995821857444388
Nerve_Tibial	-1.07373385449799e-10	5.02387440222845e-10	0.58461973152319	0.995821857444388
Pituitary	-1.24942247082212e-10	4.82374713908724e-10	0.602188134951389	0.995821857444388
Adipose_Subcutaneous	-1.22624000576688e-10	4.28467217878571e-10	0.612634563464855	0.995821857444388
Adrenal_Gland	-2.2961243570084e-10	5.26366875929067e-10	0.668661907362215	0.995821857444388
Artery_Tibial	-2.60211458565785e-10	5.23087472824966e-10	0.69056519812001	0.995821857444388
Muscle_Skeletal	-2.26237508099111e-10	4.00787410147794e-10	0.713787121162528	0.995821857444388
Vagina	-3.3161046196716e-10	4.32674781060627e-10	0.778286683070639	0.995821857444388
Pancreas	-4.14757530108448e-10	5.17991766650864e-10	0.788348179740511	0.995821857444388
Esophagus_Mucosa	-4.96558970992338e-10	5.55234542039996e-10	0.814425427034834	0.995821857444388

Name	Coefficient	Coefficient_std_error	Coefficient_P_value	FDR
Brain_Hypothalamus	-3.14289110220544e-10	3.47320984108962e-10	0.817239559274993	0.995821857444388
Liver	-4.8271905362485e-10	5.18736014682292e-10	0.823961422122682	0.995821857444388
Brain_Putamen_(basal_ganglia)	-3.74676199714351e-10	3.4440473272895e-10	0.861679276488704	0.995821857444388
Brain_Amygdala	-3.65473624605292e-10	3.33767666796653e-10	0.863240388496142	0.995821857444388
Artery_Coronary	-7.27558338979954e-10	5.92277059104703e-10	0.890353223015359	0.995821857444388
Brain_Cortex	-4.91734138424096e-10	3.53443696722932e-10	0.917927584418826	0.995821857444388
Brain_Frontal_Cortex_(BA9)	-5.00616642998226e-10	3.41724877183556e-10	0.928535428480291	0.995821857444388
Brain_Caudate_(basal_ganglia)	-5.26497186456481e-10	3.490560143963e-10	0.934266981317529	0.995821857444388
Brain_Substantia_nigra	-5.01641791263432e-10	3.29947906372047e-10	0.935790573589004	0.995821857444388
Brain_Anterior_cingulate_cortex_(BA24)	-5.66203166468338e-10	3.53273192490496e-10	0.94550336868208	0.995821857444388
Brain_Cerebellum	-6.10428261440705e-10	3.68242606206128e-10	0.951308924690966	0.995821857444388
Brain_Nucleus_accumbens_(basal_ganglia)	-5.87798002130254e-10	3.5412903887376e-10	0.951526807971011	0.995821857444388
Brain_Hippocampus	-5.54790782204736e-10	3.31339679391216e-10	0.952972696360334	0.995821857444388
Heart_Left_Ventricle	-8.4964487132695e-10	4.89000843832984e-10	0.958851587821548	0.995821857444388
Brain_Cerebellar_Hemisphere	-6.82622086787739e-10	3.68715868825508e-10	0.96794038416225	0.995821857444388
Heart_Atrial_Appendage	-8.64387386779039e-10	4.33060900566748e-10	0.977032765794494	0.995821857444388
Brain_Spinal_cord_(cervical_c-1)	-1.05106836559101e-09	3.1668252185921e-10	0.999548293130857	0.999548293130857

Supplementary Table S19. Multi-tissue transcriptomic annotation enrichment results for mvCLD-associated genes.

The table reports enrichment estimates for multi-tissue transcriptomic annotations, including immune and blood-related cell or tissue categories. Annotations are ordered by ascending P value.

Interpretation. Nominal signals were dispersed across immune-related and non-respiratory annotations and none survived FDR correction; no robust transcriptomic annotation enrichment can therefore be claimed, and the immune/T-cell trends remain descriptive.

Owing to its size (205 rows × 5 columns), the complete table is provided as a separate plain-text data file: "Supplementary Table 19 - Multi-tissue transcriptomic annotation enrichment results for mvCLD-associated genes.csv". The column headers are listed below for reference.

Columns:

Name, Coefficient, Coefficient_std_error, Coefficient_P_value, FDR

Supplementary Table S20. Multi-tissue epigenomic annotation enrichment results for mvCLD-associated genes.

The table reports enrichment estimates for epigenomic annotations across tissues and cell types, including histone marks and DNase-related regulatory annotations. Annotations are ordered by ascending P value.

Interpretation. The leading nominal signals involved active enhancer and promoter marks (H3K4me1, H3K27ac) in T-helper and memory T-cell populations, but none reached FDR significance; these patterns are hypothesis-generating rather than confirmatory.

Owing to its size (489 rows × 5 columns), the complete table is provided as a separate plain-text data file: “Supplementary Table 20 - Multi-tissue epigenomic annotation enrichment results for mvCLD-associated genes.csv”. The column headers are listed below for reference.

Columns:

Name, Coefficient, Coefficient_std_error, Coefficient_P_value, FDR

Supplementary Table S21. gsMap single-cell spatial enrichment results for mvCLD in the E16.5 mouse embryo atlas.

The table provides per-location spatial coordinates, P values, $-\log_{10}(P)$ values, and anatomical annotations from the gsMap projection of mvCLD GWAS-derived signals onto the embryonic day 16.5 (E16.5) mouse embryo spatial transcriptomic atlas. The annotation-level summaries discussed in the main text were computed from the per-location P and $-\log_{10}(P)$ values. Spatial locations are ordered by ascending P value (most enriched first).

Interpretation. mvCLD genetic signals were spatially non-uniform, with the most concentrated overall enrichment in the Lung annotation (median P = 0.005), supporting the anatomical plausibility of the shared factor. Because the atlas derives from E16.5 mouse embryonic tissue, this cross-species developmental signal indicates tissue plausibility and cannot substitute for adult human diseased lung.

Owing to its size (121,767 rows × 6 columns), the complete table is provided as a separate plain-text data file: "Supplementary Table 21 - gsMap single-cell spatial enrichment results for mvCLD in the E16.5 mouse embryo atlas.csv". The column headers are listed below for reference.

Columns:
, sx, sy, p, logp, annotation

Supplementary Table *S22*. S-LDSC heritability enrichment across functional genomic annotations.

The table reports stratified LDSC estimates for functional genomic annotations, including the proportion of SNPs, proportion of SNP heritability, enrichment, coefficients, and FDR values. Annotations are ordered by ascending FDR-corrected P value, then by descending enrichment.

Interpretation. Heritability was disproportionately concentrated in regulatory genome compartments: evolutionarily conserved regions covered only 2.6% of SNPs yet explained 34.8% of mvCLD heritability (enrichment = 13.55), and transcription start sites, promoters, enhancers, super-enhancers, open chromatin, and active histone marks were significantly enriched, whereas repressed regions were depleted. This indicates that mvCLD liability acts mainly through gene-regulatory rather than protein-coding variation.

Category	Prop._SNPs	Prop._h2	Prop._h2_std_error	Enrichment	Enrichment_std_error	Enrichment_p	Coefficient	Coefficient_std_error	Coefficient_z-score	FDR
SuperEnhancer_Hnisz.extend.500.bedL2_0	0.170382974183376	0.387680800743573	0.0245642038368223	2.27534941564249	0.144170530855888	2.04274532496364e-13	-1.65156490699887e-08	1.61482559273337e-08	-1.02275125835931	1.06222756898109e-11
SuperEnhancer_Hnisz.bedL2_0	0.167209765751928	0.399226391327987	0.0258265284628476	2.38757819875352	0.154455861753695	9.67598801059526e-13	1.6382357479175e-08	1.62598212273725e-08	1.00753613770342	2.51575688275477e-11
Repressed_Hoffman.extend.500.bedL2_0	0.718510611778683	0.406337645946393	0.0341785853375481	0.56552768920211	0.047568657688908	3.03149892972052e-12	7.41599745279408e-10	1.71130895620393e-09	0.433352342714574	5.2545981448489e-11
Conserved_LindbladToh.bedL2_0	0.0256851058661579	0.348111522107217	0.0491732998506946	13.5530499239982	1.91446747803692	2.29355533340738e-11	4.35225467759745e-08	7.07870788929092e-09	6.14837445712627	2.38529754674367e-10
H3K27ac_Hnisz.bedL2_0	0.38907584917631	0.744154146385191	0.0372637348586997	1.91261973201523	0.0957749881869787	1.85120272914197e-11	2.83587278589737e-09	4.86246687033339e-09	0.583216885897863	2.38529754674367e-10
H3K27ac_PGC2.extend.500.bedL2_0	0.335206459012417	0.69646770704224	0.04581403802761	2.07772758643783	0.136674090835204	2.89337750337689e-10	-5.68485415705978e-09	3.79675646631124e-09	-1.49729228290034	2.50759383625997e-09
H3K4me1_Trynka.extend.500.bedL2_0	0.605819103298536	0.890036510105931	0.0468879888496553	1.46914566618963	0.0773960223346569	5.86585873431068e-09	-1.31006114706586e-09	2.23928538647009e-09	-0.585035366631398	4.35749505977365e-08
H3K27ac_PGC2.bedL2_0	0.268777933955461	0.764148255398527	0.0746883566760205	2.84304683852936	0.277881281312316	1.00217403256249e-08	8.5090167385629e-09	5.07216325795326e-09	1.6775912575804	6.51413121165621e-08
FetalDHS_Trynka.extend.500.bedL2_0	0.283361842889948	0.789479299326307	0.0729479879857729	2.78611718244974	0.257437583133253	2.45989312039799e-08	3.45298224229684e-09	2.69168075640689e-09	1.28283498482421	1.36359439903663e-07
H3K9ac_Trynka.extend.500.bedL2_0	0.229854798370585	0.581761618863348	0.0573243140465335	2.53099618971364	0.249393593054829	2.62229692122428e-08	-5.64679313403898e-10	3.68181532236325e-09	-0.153369809173766	1.36359439903663e-07
H3K27ac_Hnisz.extend.500.bedL2_0	0.420486351731266	0.735828497683771	0.0428249499558596	1.74994621027329	0.101846230631593	6.17511584288186e-08	-2.60835819883448e-09	4.99251645041319e-09	-0.522453601253254	2.91914567118052e-07
H3K4me3_Try	0.1330105437	0.5339396916	0.0609987348	4.0142659111	0.4586007479	3.0282282474	7.3305000521	4.3676106005	1.6783776582	1.31223224058

Category	Prop._SNPs	Prop._h2	Prop._h2_std_error	Enrichment	Enrichment_std_error	Enrichment_p	Coefficient	Coefficient_std_error	Coefficient_z-score	FDR
nka.bedL2_0	54998	18148	490388	4198	33163	9505e-07	2778e-09	5986e-09	9859	119e-06
H3K4me3_Trynka.extend.500.bedL2_0	0.255139143243789	0.598652581424762	0.0574302000592107	2.34637685857847	0.225093646271029	5.77479508884945e-07	-2.61285743412307e-09	2.56955413302957e-09	-1.01685245721694	2.30991803553978e-06
TSS_Hoffman.extend.500.bedL2_0	0.0343533866484689	0.230938115140639	0.0334493890641441	6.72242645255854	0.973685343061656	6.49629228114828e-07	-2.00640677040917e-09	6.63989220192833e-09	-0.302174600037404	2.41290856156936e-06
TFBS_ENCODE.extend.500.bedL2_0	0.341214518854471	0.830446866819727	0.0757628956195118	2.43379698380864	0.222038897623301	1.04693399478748e-06	1.29322122178518e-09	2.36218280645688e-09	0.547468730299042	3.62937118192992e-06
Conserved_LindbladToh.extend.500.bedL2_0	0.330412424832151	0.736852368687687	0.0752562511957719	2.23009885013255	0.227764592188087	2.05215786307511e-06	2.65269025644068e-10	1.47569295807405e-09	0.179758956084113	6.66951305499411e-06
H3K4me1_Trynka.bedL2_0	0.423684555302081	0.873774378975844	0.0948980294871524	2.06232294295659	0.223982744472456	4.58371312257453e-06	2.12434103709176e-09	3.34657914474921e-09	0.634779858837302	1.40207695514044e-05
DHS_Trynka.extend.500.bedL2_0	0.496042128720271	0.862552759933561	0.0817474913600116	1.73886996686923	0.164799493081183	1.25938852271947e-05	-2.7906084068001e-09	2.95716416704294e-09	-0.943677201929108	3.63823351007847e-05
Enhancer_Hoffman.extend.500.bedL2_0	0.0898451123346987	0.407912678966138	0.059101279001211	4.54017662581963	0.657812956825541	1.54612066865341e-05	2.10511631456916e-09	5.89916857826639e-09	0.356849662226096	4.23148814578827e-05
Intron_UCSC.extend.500.bedL2_0	0.396796495446607	0.499440666627861	0.0223590838672118	1.2586821515793	0.0563489953257927	2.54313652533647e-05	-2.07411268689364e-08	1.26244122781074e-08	-1.64293801660015	6.61215496587481e-05
Enhancer_Andersson.extend.500.bedL2_0	0.0190350567733557	0.14600781589345 8	0.0281561058369433	7.67046915761409	1.47917109847315	3.16607962401002e-05	8.15317975865008e-09	7.9235808620566e-09	1.02897665848189	7.83981621183434e-05
FetalDHS_Trynka.bedL2_0	0.0839308262034279	0.439865770480707	0.0758345166050703	5.24081306449408	0.903535923991334	3.6955979500307e-05	2.85626468948236e-09	6.93882198689434e-09	0.41163538924577	8.73504970007257e-05
H3K9ac_Trynka.bedL2_0	0.125412860150182	0.436280238242243	0.0687127288601157	3.47875200134816	0.547892208006675	5.79768290760653e-05	-4.21493140508146e-09	5.93952128376384e-09	-0.709641603036817	0.000131078048345887
DHS_Trynka.bedL2_0	0.166200566030868	0.616216056953754	0.10477593032067	3.70766521239951	0.630418613022118	0.000101664253549262	-1.81082504892682e-09	5.38641255593413e-09	-0.336183875654281	0.000220272549356735
DGF_ENCODE.extend.500.bedL2_0	0.53847028740552	0.955322833938587	0.0987017035092699	1.77414215098397	0.183300185391544	0.000107679079243256	7.08956091947739e-10	2.94809581258055e-09	0.240479325306314	0.000223972484825972
TSS_Hoffman.bedL2_0	0.0178321027840391	0.18157523948 6	0.0405523500582151	10.1824917501329	2.27412047526509	0.000141343161575556	1.86106413041355e-08	1.43993998957616e-08	1.29245950795584	0.000282686323151111

**Category**	**Prop._SNPs**	**Prop._h2**	**Prop._h2_std_error**	**Enrichment**	**Enrichment_std_error**	**Enrichment_p**	**Coefficient**	**Coefficient_std_error**	**Coefficient_z-score**	**FDR**
TFBS_ENCODE.bedL2_0	0.13122817223966	0.549808452979309	0.109807525629981	4.18971356223115	0.83676792685523	0.000272668123081219	1.30003154446874e-09	5.45269998913091e-09	0.238419782320713	0.000525138607415681
Coding_UCSC.bedL2_0	0.0142749086209578	0.0896730662292103	0.0217441856957166	6.28186621787241	1.52324517607018	0.000326507278098986	7.10476006174287e-09	7.44322044456767e-09	0.954527695995915	0.000606370659326689
Promoter_UCSC.extend.500.bedL2_0	0.0569345659124342	0.157806521313549	0.0260031700158704	2.77171730010652	0.456720264730982	0.000426007207347599	-3.12161414103626e-08	1.52317082457932e-08	-2.04941828629	0.000763874992485349
DGF_ENCODE.bedL2_0	0.136005598911218	0.615909204090939	0.134486993668025	4.52855771395846	0.988834244653527	0.000547847130357013	8.07292452114877e-09	5.53183679635882e-09	1.45935695833661	0.000949601692618823
WeakEnhancer_Hoffman.extend.500.bedL2_0	0.0887865933453545	0.28834913257228	0.0600793944761926	3.24766523534341	0.676671918726523	0.00112999148738526	-1.14096646244178e-09	4.79886551007146e-09	-0.237757540828601	0.00189546959174301
Promoter_UCSC.bedL2_0	0.0463455177088885	0.2288297132282	0.0508429419636877	4.93747237145035	1.09704119140602	0.00184756181451423	3.54742908533179e-08	1.78640904370299e-08	1.985787688344	0.00300228794858562
Enhancer_Hoffman.bedL2_0	0.0419891500964829	0.266494245094265	0.0653670363070041	6.34674063375689	1.5567601667765	0.00196076224531539	7.09171713175411e-09	1.08284080902167e-08	0.654917793333019	0.00302397437081189
H3K4me1_peaks_Trynka.bedL2_0	0.169822512702647	0.438605340442777	0.0870502342131363	2.58272789315489	0.51259537282644	0.00197721401168469	-3.38991180779411e-09	3.22259485780039e-09	-1.05191994568871	0.00302397437081189
DHS_peaks_Trynka.bedL2_0	0.110582858132118	0.492335144651718	0.115090426922784	4.45218321327437	1.04076191253151	0.00259157389481376	7.46216505508544e-10	7.78953383910078e-09	0.0957973250931646	0.00383037244947301
Coding_UCSC.extend.500.bedL2_0	0.0636617812073122	0.236757311177613	0.0506559151105386	3.71898659898664	0.795703704010095	0.00265179631117362	3.10579029687055e-09	4.66488401890734e-09	0.665780817761472	0.00383037244947301
UTR_5_UCSC.extend.500.bedL2_0	0.0268650777474649	0.154709202562103	0.0463958124790197	5.75874762084773	1.72699341930614	0.0103043447318738	6.60189862533945e-09	6.27926156640621e-09	1.05138136953226	0.0144817817853362
WeakEnhancer_Hoffman.bedL2_0	0.0209871939332603	0.156657893075462	0.0547907058641801	7.46445158765087	2.610673253338	0.0129186358152667	1.56702938839179e-08	1.15770062896254e-08	1.35357047339264	0.0176781332208912
Intron_UCSC.bedL2_0	0.387472972957105	0.454992895272902	0.0267884804549039	1.1742571147621	0.0691363845340242	0.0168906678456359	2.0813542964576e-08	1.26064886965817e-08	1.65101825460881	0.0225208904608479
Enhancer_Andersson.bedL2_0	0.00432768191554696	0.0712187671402061	0.0267533078662211	16.4565623190458	6.18190254928657	0.0194521491721648	3.35367029742822e-08	2.82249556154929e-08	1.18819329359277	0.0252877939238143
UTR_3_UCSC.extend.500.bedL2_0	0.0264552581133971	0.0827143134564948	0.0240231362401858	3.12657367023034	0.908066598224583	0.0200335168596939	2.47269904337172e-10	5.9706058944953e-09	0.0414145412888744	0.0254083628464411
H3K9ac_peaks_Trynka.bedL2	0.0384594673619677	0.208145095839468	0.0805825892789274	5.41206392383117	2.09526014805434	0.0314090247406795	6.13895020837473e-09	1.0600727749388e-08	0.579106487168216	0.0379830066631473

Category	Prop._SNPs	Prop._h2	Prop._h2_std_error	Enrichment	Enrichment_std_error	Enrichment_p	Coefficient	Coefficient_std_error	Coefficient_z-score	FDR
_0										
Repressed_Hoffman.bedL2_0	0.460873296618996	0.241035067379502	0.100183660225925	0.522996383491416	0.217377880126448	0.0306954369141283	9.11736694278606e-10	2.26408556428773e-09	0.40269533477876	0.0379830066631473
CTCF_Hoffman.extend.500.bedL2_0	0.0708357217111639	0.248850860961797	0.0802664547400777	3.51307017067601	1.13313527131647	0.0386509307403401	9.75611689298706e-09	7.19560693821681e-09	1.35584349961795	0.0456783726931293
H3K4me3_peaks_Trynka.bedL2_0	0.0416487800442833	0.160811412721455	0.0638164328802485	3.86113140770201	1.5322521527016	0.0623024873356932	-8.94015831052651e-09	9.1115020609019e-09	-0.981194785532604	0.0704288987273054
UTR_3_UCSC.bedL2_0	0.0111859120013407	0.0408955317826301	0.0167615599955319	3.65598547331041	1.49845269599143	0.0615993273736919	1.07719803094995e-10	9.34471543571235e-09	0.0115273497450042	0.0704288987273054
Transcribed_Hoffman.extend.500.bedL2_0	0.762095256979389	0.680646640491258	0.0447908647143356	0.893125412155224	0.0587733151520545	0.0699263813023868	1.11393892915394e-09	1.43625555328672e-09	0.775585463606952	0.0773653580366833
PromoterFlanking_Hoffman.extend.500.bedL2_0	0.0331864994709921	0.103260568791699	0.0427550717569244	3.11152337359227	1.28832725471079	0.110701297254705	-2.7267552920604e-09	8.62562922565906e-09	-0.316122478804096	0.119926405359263
UTR_5_UCSC.bedL2_0	0.00548416843100478	0.038635097408669	0.0254798861846243	7.04484150965263	4.64608016788355	0.19348542422721	2.52729801032654e-09	1.73595884174403e-08	0.145585134252808	0.205331470608467
Transcribed_Hoffman.bedL2_0	0.345967789149727	0.28259555998184	0.0699328186575683	0.816826215747901	0.202136790911777	0.370565361843099	-5.54518505494116e-10	1.62292397115873e-09	-0.34167867093503	0.385387976316823
PromoterFlanking_Hoffman.bedL2_0	0.00830845142697922	0.0296902023976104	0.0254705328274471	3.57349412926702	3.06561734774535	0.39789298913588	4.60230271697434e-09	1.82408755516521e-08	0.25230711672486	0.405694813143267
CTCF_Hoffman.bedL2_0	0.0238661642811406	0.0590106011040465	0.0516169934758112	2.47256326609121	2.16276871590127	0.495767255764782	-8.65954112755542e-09	1.18221096119462e-08	-0.732486959755897	0.495767255764782
baseL2_0	1	1	2.82253743348489e-08	1	2.82253743348489e-08	NA	-2.10956071295717e-09	1.74652206402127e-09	-1.20786376331257	NA

Supplementary Table S23. Chromosome-level aggregation of PRS-CS posterior effect-size weights.

The table summarizes the number of SNPs and the signed aggregate posterior effect-size weight for each autosome based on PRS-CS posterior SNP effect estimates. Values represent signed aggregate posterior effect-size weights and should not be interpreted as validated risk prediction metrics or chromosome-specific heritability estimates. Chromosomes are ordered by descending absolute signed posterior effect-size weight.

Interpretation. This table provides a descriptive genome-wide summary of polygenic weight distribution across the 22 autosomes (897,970 SNPs); the signed per-chromosome sums (largest positive on chromosome 9, largest negative on chromosome 18) reflect the aggregate direction of posterior effect sizes and are not validated risk-prediction metrics or chromosome-specific heritability estimates.

Chromosome	N_SNPs	Signed Weight Sum
chr18	27217	-0.134929560936
chr2	75750	-0.125655170700
chr1	75709	-0.122834489953
chr20	23848	-0.118740582445
chr11	46212	-0.104766577267
chr4	56475	-0.100015999604
chr9	41372	0.096648788008
chr3	63012	-0.090923071226
chr8	47921	-0.067359975926
chr12	44950	-0.050013729320
chr7	49530	0.047286324262
chr15	27073	0.045735313766
chr14	30186	-0.043607124302
chr5	57641	-0.042971833097
chr13	34625	-0.042232881317
chr6	54874	-0.031012009092
chr17	23926	-0.025532283986
chr21	12694	-0.023259844869
chr10	48586	-0.021789901154
chr22	13311	0.017979348941
chr19	16685	0.004152034174
chr16	26373	-0.001706788179

Abbreviations

Abbreviation	Definition
AIC	Akaike information criterion
CFI	comparative fit index
CLD	chronic lung disease
COPD	chronic obstructive pulmonary disease
E16.5	embryonic day 16.5
FDR	false discovery rate
FINEMAP	Bayesian fine-mapping method
FOCUS	Fine-mapping Of CaUsal gene Sets
FUMA	Functional Mapping and Annotation of Genome-Wide Association Studies
GWAS	genome-wide association study
GTEx	Genotype-Tissue Expression
IPF	idiopathic pulmonary fibrosis
LD	linkage disequilibrium
LDSC	linkage disequilibrium score regression
MAGMA	Multi-marker Analysis of GenoMic Annotation
MAF	minor allele frequency
mvCLD	multivariate chronic lung disease
PIP	posterior inclusion probability
PRS	polygenic risk score
PRS-CS	polygenic risk score-continuous shrinkage
S-LDSC	stratified linkage disequilibrium score regression
SEM	structural equation modeling
SNP	single nucleotide polymorphism
SRMR	standardized root mean square residual
SuSiE	Sum of Single Effects
TWAS	transcriptome-wide association study
